# Seeing Gravity: Gait Adaptations to Visual and Physical Inclines – A Virtual Reality Study

**DOI:** 10.3389/fnins.2019.01308

**Published:** 2020-01-24

**Authors:** Desiderio Cano Porras, Gabriel Zeilig, Glen M. Doniger, Yotam Bahat, Rivka Inzelberg, Meir Plotnik

**Affiliations:** ^1^Center of Advanced Technologies in Rehabilitation, Sheba Medical Center, Ramat Gan, Israel; ^2^Sackler Faculty of Medicine, Tel Aviv University, Tel Aviv, Israel; ^3^Perception and Action in Complex Environments, Marie Curie International Training Network, European Union’s Horizons 2020 Research and Innovation Program, Brussels, Belgium; ^4^Department of Neurological Rehabilitation, Sheba Medical Center, Ramat Gan, Israel; ^5^Department of Physical and Rehabilitation Medicine, Sackler Faculty of Medicine, Tel Aviv University, Tel Aviv, Israel; ^6^Department of Clinical Research, NeuroTrax Corporation, Modiin, Israel; ^7^The Joseph Sagol Neuroscience Center, Sheba Medical Center, Ramat Gan, Israel; ^8^Sagol School of Neuroscience, Tel Aviv University, Tel Aviv, Israel; ^9^Department of Neurology and Neurosurgery, Sackler Faculty of Medicine, Tel Aviv University, Tel Aviv, Israel; ^10^Department of Applied Mathematics and Computer Science, The Weizmann Institute of Science, Rehovot, Israel; ^11^Department of Physiology and Pharmacology, Sackler Faculty of Medicine, Tel Aviv University, Tel Aviv, Israel

**Keywords:** virtual reality, perception and action, sensorimotor integration, gravity, locomotion, vision, multisensory integration

## Abstract

Using advanced virtual reality technology, we demonstrate that exposure to virtual inclinations visually simulating inclined walking induces gait modulations in a manner consistent with expected gravitational forces (i.e., acting upon a free body), suggesting vision-based perception of gravity. The force of gravity critically impacts the regulation of our movements. However, how humans perceive and incorporate gravity into locomotion is not well understood. In this study, we introduce a novel paradigm for exposing humans to incongruent sensory information under conditions constrained by distinct gravitational effects, facilitating analysis of the consistency of human locomotion with expected gravitational forces. Young healthy adults walked under conditions of actual physical inclinations as well as virtual inclinations. We identify and describe ‘braking’ and ‘exertion’ effects – locomotor adaptations accommodating gravito-inertial forces associated with physical inclines. We show that purely visual cues (from virtual inclinations) induce consistent locomotor adaptations to counter expected gravity-based changes, consistent with *indirect prediction* mechanisms. Specifically, downhill visual cues activate the braking effect in anticipation of a gravitational boost, whereas uphill visual cues promote an exertion effect in anticipation of gravitational deceleration. Although participants initially rely upon vision to accommodate environmental changes, a sensory reweighting mechanism gradually reprioritizes body-based cues over visual ones. A high-level neural model outlines a putative pathway subserving the observed effects. Our findings may be pivotal in designing virtual reality-based paradigms for understanding perception and action in complex environments with potential translational benefits.

## Introduction

Gravity greatly influences human walking (Cavagna et al., 2000). For example, locomotion on inclined planes involves sensing and accommodating gravitational forces (Saibene and Minetti, 2003; Lacquaniti et al., 2014; Maffei et al., 2015). While uphill walking requires more effort to counteract gravity, humans typically apply resistance against the associated gravitational boost during downhill walking (Saibene and Minetti, 2003; Hunter et al., 2010; Kimel-Naor et al., 2017). Inclined planes also affect energy expenditure during walking (Margaria, 1976; Saibene and Minetti, 2003), with uphill walking usually requiring twice the oxygen consumption and producing 50% more heat than downhill walking (Johnson et al., 2002). Thus, optimal sensorimotor integration on inclined planes inherently incorporates the anticipated modulatory effects of gravity (Lacquaniti et al., 2014, 2015; Maffei et al., 2015). Locomotion is also a visually guided behavior – available visual cues govern gait adaptations to new environments (Zhao and Warren, 2015; Barton et al., 2017). How sensorimotor integration incorporates perception of gravity and visual cues during locomotion, however, is not fully understood.

To investigate the roles of vision and gravity on locomotor adaptation, we devised a paradigm involving *virtual inclinations* – a virtual reality (VR) environment simulating uphill and downhill walking. Previous studies suggest that manipulating VR-generated visual feedback modulates gait in healthy adults (Mohler et al., 2007; O’Connor and Donelan, 2012; Thompson and Franz, 2017) as well as in individuals with conditions characterized by gait impairment like stroke (Lamontagne et al., 2007; Ala’S and Lamontagne, 2013) and Parkinson’s disease (van Wegen et al., 2006; Zhao et al., 2016). Most studies introduced a mismatch between VR-generated visual flow velocity (*virtual velocity*) and actual (i.e., treadmill) walking speed. The strategy adopted by healthy participants was to reduce walking speed at faster virtual velocities and to increase walking speed at slower virtual velocities (Prokop et al., 1997; Mohler et al., 2007; Guerin and Bardy, 2008).

**FIGURE 1:**
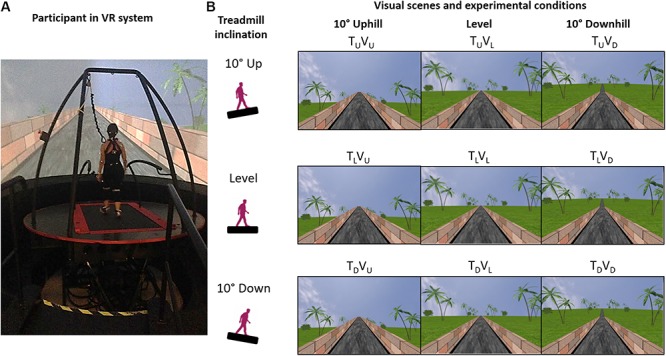
Apparatus and experimental conditions. **(A)** A fully immersive virtual reality (VR) system containing an embedded treadmill synchronized with visual scenes projecting a moving road on a large 360° dome-shaped screen in a room-sized VR facility. **(B)** During level walking, participants experienced nine different conditions presented in random order, in which the inclination of the treadmill (T) and/or visual scenes (V) transitioned to 10° uphill (U), remained level at 0° (L) or transitioned to -10° downhill (D). Conditions are illustrated as a 3 × 3 matrix, in which rows represent the inclination of the treadmill (T) and columns represent the inclination of the visual scene (V). Congruent condition T_L_V_L_ represents continued level walking, and T_U_V_U_ and T_D_V_D_ represent congruent uphill and downhill walking, respectively. The other conditions are incongruent: only vision up (T_L_V_U_) or down (T_L_V_D_), only treadmill up (T_U_V_L_) or down (T_D_V_L_), treadmill down-vision up (T_D_V_U_), and treadmill up-vision down (T_U_V_D_).

As these studies demonstrated visual modulation of locomotion during level walking alone, they were unable to elucidate the mechanisms for incorporating gravitational effects under real-life walking conditions (Barton et al., 2017) characterized by both level *and inclined* walking, which involve a variety of gravitational forces (Hollerbach et al., 2001). To address this gap, we designed a study in which healthy participants walk on a self-paced treadmill synchronized with a visual scene projected in a VR facility (Plotnik et al., 2013, 2015) (Figure 1A). Our study design enabled us to dissociate the impact of visual vs. physical body-based cues by testing conditions in which the inclination of the visual scene was either congruent or incongruent with the physical inclination of the treadmill (Figure 1B). Thus this paradigm allowed us to effectively disrupt the dynamics of perception and action (Thompson et al., 2005; Warren, 2006).

To help account for changes in locomotion induced by our paradigm, we invoke models of sensorimotor integration (O’Connor and Donelan, 2012). The *indirect prediction* model states that neural mechanisms controlling locomotion (e.g., central pattern generators) and reliance on accumulated experience, promptly activate pre-programmed gait patterns following destabilizing environmental changes (Pearson, 2004; Snaterse et al., 2011; O’Connor and Donelan, 2012). Then there is a recalibration of the relative influence of visual and body-based cues leading to gradual re-stabilization of walking patterns: an iterative mechanism known as *sensory reweighting* (O’Connor and Donelan, 2012; Campos et al., 2014; Assländer and Peterka, 2016).

We hypothesized that the mechanisms of *indirect prediction* and *sensory reweighting* would govern locomotor adaptation in our study. Upon exposure to visual information incongruent with the physical inclination of the treadmill, we anticipated two scenarios. First, in keeping with *indirect prediction*, incongruent uphill inclinations of the visual scene would initially accelerate walking because the uphill visual cues would induce expenditure of additional energy, as required to counteract gravity during natural uphill walking (*exertion effect*). Conversely, incongruent downhill inclinations of the visual scene would initially decelerate walking, to counteract the anticipated (gravitational) boost that occurs during natural downhill walking (*braking effect*). Then, in keeping with *sensory reweighting*, we predicted gradual adaptation to the discordant visual feedback as body-based cues (consistent with physical inclination of the treadmill) rather than visual ones begin to guide locomotion.

## Materials and Methods

### Participants

Sixteen young healthy adults (mean age ± 1 SD: 27.25 ± 3.85 years, 9 female) participated in this study. None of the participants had cognitive limitations, physical restrictions or sensorimotor impairments that could potentially affect locomotion or the ability to adhere to instructions. We excluded one participant when it became apparent that he was not naïve to the experimental design (i.e., participants were asked to give open-ended feedback following the experimental session, and only this participant reported intentionally adjusting gait in anticipation of incongruent visual cues throughout the session). The Institutional Review Board for Ethics in Human Studies at the Sheba Medical Center, Israel, approved the experimental protocol, and all participants gave written informed consent before being enrolled in the study.

### Apparatus

Experiments were conducted with a fully immersive virtual reality system (CAREN High End, Motek Medical, Netherlands; Figure 1A) containing a moveable platform with six degrees of freedom (Kimel-Naor et al., 2017). The platform contained an embedded treadmill that operated in self-paced mode, allowing participants to adjust treadmill speed to preferred walking speed (Plotnik et al., 2015). Walking speed was estimated directly from a tachometer in the treadmill motor that provides the velocity signals from the treadmill belts. Simultaneously, a motion capture system (Vicon, Oxford, United Kingdom) tracked the three-dimensional coordinates of 41 passive markers affixed to the body of each participant with a sampling rate of 120 Hz and spatial accuracy of 1 mm. The implemented marker set-up followed Vicon’s ‘HumanRTKM’ model.

### Visual Stimuli

Visual scenes simulated walking on an asphalt road in a park, with a brick wall on either side of the road and greenery adjacent to the wall (Figure 1). Previous to each experiment, we verified that each participant could clearly see the visual scenery of the virtual environment. In the ‘vision level’ conditions (Figure 1B, middle column), the brick wall ends at the horizon. In the ‘vision up’ conditions, the wall ends above the horizon (Figure 1B, left column), and in the ‘vision down’ conditions, the wall ends below the horizon (Figure 1B, right column). Scenes were modeled in three dimensions with specialized software (Autodesk XSI). Textures were created and modified with Adobe Photoshop. Custom software (D-Flow, Motek Medical, Netherlands) was used for programing, integration and projection, as well as for moving/rotating the platform and activating the treadmill. A virtual camera was placed in the virtual world, in a position representing the center of the lab, which is also the center of the moveable platform. A treadmill was embedded on the platform. During the experimental session, the visual scene advanced (i.e., visual flow) at a speed synchronous with the speed of the treadmill (anteroposterior axis, operated in self-paced mode) – the virtual camera was stationary and the entire virtual world moved around it. The visual scenes were projected on a 360° dome-shaped screen (six meters in diameter) by eight video projectors. Projector resolution was 1400 × 1050 pixels, and participant viewing distance was 3 m.

### Habituation Period

After calibrating the motion capture system, the participant was familiarized with the system and the treadmill self-paced mode during a short habituation period (10–15 min.). Familiarization comprised an initial stage to master self-paced mode during level walking followed by a second stage to practice walking in all congruent conditions (i.e., level, uphill, downhill). Afterward, we exposed the participants to all nine experimental conditions (Figure 1B) in random order. The instructions were to walk “normally at your most comfortable pace” and that “inclinations may be introduced while you are walking.”

### Experimental Conditions (See Figure 1B)

Inclinations of the treadmill and the visual scenes were of 10°, we increased by 1° the 9° limit for road inclines in North America (Proffitt et al., 1995). T_L_V_L_: congruent level walking; T_U_V_U_: congruent uphill walking; T_D_V_D_: congruent downhill walking; T_L_V_U_: only vision up; T_L_V_D_: only vision down; T_U_V_L_: only treadmill up; T_D_V_L_: only treadmill down; T_D_V_U_: vision up-treadmill down; T_U_V_D_: vision down-treadmill up.

### Procedure

During the experimental session, participants began walking with both treadmill and the visual scene level. Transition of the inclination of the treadmill and/or visual scene (except in the T_L_V_L_ condition) began when the participant reached a ‘steady-state velocity’ (see below). Transition period was 5 s. Participants walked for 70 s post-transition in each condition (i.e., from transition initiation); data from the first minute was analyzed. By convention, we refer to the transition start time as time zero (*t* = 0).

### Steady-State Velocity

A real-time algorithm monitoring treadmill speed determined steady-state velocity (ssv). According to the algorithm, ssv is attained after: (1) a minimum 30 s of walking, and (2) a consecutive period of 12 s with walking speed coefficient of variance less than two percent. Upon satisfying both conditions, transition of treadmill and/or visual scene inclination (as appropriate for the experimental condition) was automatically triggered. We defined onset time of the effect as the moment in which walking speed goes beyond the mean ± 2 SD from the ssv (i.e., defined by the 12 s period).

### Normalization of Walking Speed

**FIGURE 2:**
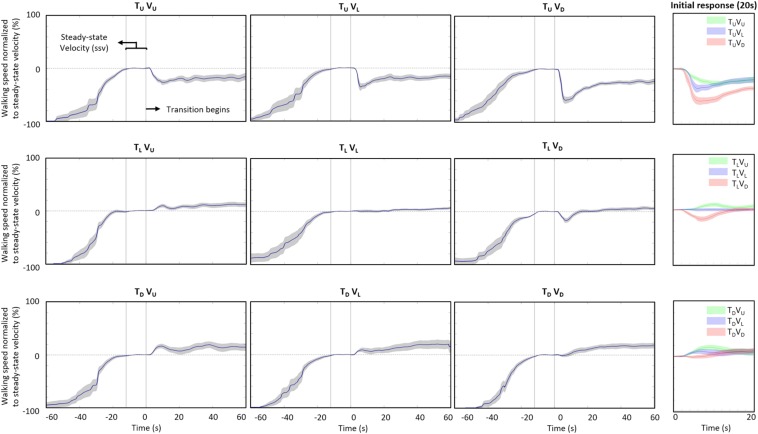
Adaptation of walking speed. Average self-paced walking speed (15 participants) relative to steady-state velocity for each condition (see values in Supplementary Table S1). Data is shown in a 3 × 3 matrix, with rows representing inclination of the treadmill (T) and columns inclination of the visual scene (V) upon transition (of the treadmill and/or visual scene) from its original level (L) inclination to a 10° uphill (U) or downhill (D) inclination. No transition occurred for the T_L_V_L_ condition. Time zero marks the end of the steady-state velocity period, after which the transition occurred for 5 s. Gray shading represents the standard error. To facilitate comparison, the rightmost column shows magnified data for each treadmill inclination for the 20 s post-transition, with data for all visual scene inclinations plotted on the same graph (U: green; L: blue; D: red). Irrespective of treadmill inclination upon transition, there is a tendency for downward visual transition to decrease walking speed and, to a lesser degree, for upward visual transition to increase walking speed.

To compare effects across conditions, we normalized walking speed, which was our primary outcome. Normalization of walking speed (WS) in each experimental condition consisted of three steps. First, WS was divided by average ssv (i.e., from the 12 s that defined the ssv period). Second, we subtracted one from the resulting value to set normalized WS to approximately zero at *t* = 0. Lastly, to obtain percent values, we multiplied by 100. Therefore, normalized WS represents the percent change in WS relative to ssv (Figure 2). Supplementary Table S1 shows the ssv values for the nine experimental conditions.

(1) $$N\,o\,r\,m\,a\,l\,i\,z\,e\,d\,W\,S=\left(\frac{W\,S}{s\,s\,v}-1\right)*100$$

### Calculation of Exertion Effect and Braking Effect (See Figure 3A)

To elucidate gravity effects, we compared walking behavior to the effect of gravity on a free body. A free body moving along an inclined plane follows this primary kinematic equation:

**FIGURE 3:**
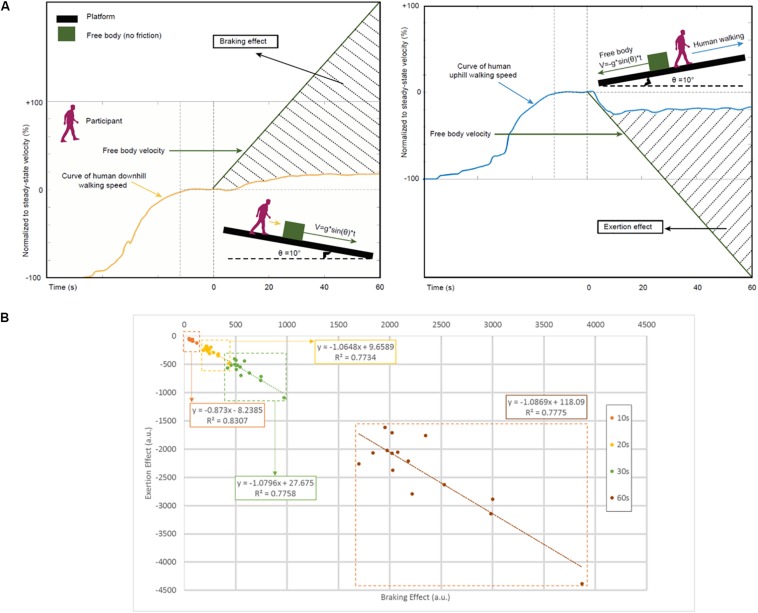
The relation of locomotion and gravity while walking in inclined planes. **(A)** Schematic characterization of the braking (left panel) and exertion (right panel) effects. We introduce a new measure for estimating braking (positive values) and exertion (negative values) effects by calculating the shaded area using numerical integration (Supplementary Figure S1 and section Materials and Methods). Free body velocity was rescaled to permit graphical comparison on the same scale as the walking data. **(B)** Relationship between braking effect and exertion effect. Scatterplots and linear regression lines for the relationship between estimated braking and exertion effects in study participants included separate time windows covering the entire experimental period: at 10 s (orange), 20 s (yellow), 30 s (green), and 60 s (brown) post-transition from steady-state velocity. Regression equations and r-squared values are shown. The correlations represented in these data suggest similar volitional effort to counteract the natural tendencies imposed by gravity in uphill and downhill walking. a.u., arbitrary units.

(2) $$V\,\left(t\right)=V_{o}+a\,t$$

where *V*_*o*_ and *V*(*t*) are initial and instantaneous velocities, respectively, *a* is acceleration and *t* is time. To obtain acceleration, we apply net forces formulas in inclined planes (equations 3 and 4). We can define the force accelerating the body parallel to the incline as *F*_*g*_ and the force opposing that motion due to friction as *F*_*f*_. If we define the direction of motion as axis “x”, the net force in the x direction is:

(3) $$\sum F_{x}=F_{g}-F_{f}$$

We can apply inclined plane kinematics to rewrite equation (2) as follows:

(4) $$m\,a=m\,g\,\sin\theta -\mu_{f}\,F_{N}$$

where *m* is mass, μ_*f*_ is the coefficient of friction and *F*_*N*_ is the normal force. Assuming no friction and initial velocity zero, the acceleration is equal to *gsin*⁡θ and a combination of equations (2) and (4) allows estimating the velocity of a free body over time as follows:

(5) V⁢(t)=g*sin⁢θ*t

where θ is the angle of inclination (i.e., 10° in our study) and *g* is gravitational acceleration (i.e., ∼9.8 m/s^2^).

We compared actual WS with the theoretical velocity of a free body moving on an inclined plane *V*(*t*)over time by calculating the area under the curve (AUC) of the delta between these two signals [i.e., Δ(*V*(*t*)−*WS*), with both normalized to ssv]. We used numerical integration for calculating AUC. Negative values are possible as, when Δ(*V*(*t*)−*WS*) is negative, the AUC will be as well. This case applies to calculation of the exertion effect in uphill walking, whereby gravity decelerates walking. Thus whether the sign is positive or negative reflects whether gravity effects are acceleratory (positive) or deceleratory (negative).

*Braking effect* is computed as AUC for Δ(*V*(*t*)−*WS*) for *V*(*t*)with θ = 10° and WS from congruent downhill walking (condition T_D_V_D_). *Exertion effect* is computed as AUC using the same equation for *V*(*t*)with θ = −10° and WS from congruent uphill walking (condition T_U_V_U_).

### Calculation of Area Under the Curve in Experimental Conditions

The calculation of AUC for (1) braking effect, (2) exertion effect, (3) *V*(*t*) and (4) *WS* derived from the experimental conditions, involved numerical integration over specific regions of interest (e.g., at 5, 10, or 60 s). Supplementary Figure S1 illustrates the calculation of AUC for *WS* related to uphill and downhill visual transitions.

### Calculation of Ratio of Gravity-Induced Behavior

To estimate the level of influence of gravity on WS, we calculated AUC *separately* and second-by-second (i.e., at 1 s, at 2 s… at 60 s) from *V*(*t*) and *WS* for congruent uphill and downhill walking. Then, we defined a ratio:

(6) $$R=\frac{A\,U\,C\,\left(W\,S_{i}\right)}{A\,U\,C\,\left(V\,{\left(t\right)}_{t=i}\right)}\,.100$$

The index “*i”* refers to the time (in seconds) post-transition. The ratio quantifies the degree to which *WS* approximates the velocity of a free body. A positive ratio indicates that both *WS* and a free body accelerate, or, alternatively, both decelerate in the given condition (i.e., both decelerate when uphill; both accelerate when downhill), and a negative ratio indicates that one accelerates while the other decelerates. A ratio closer to zero implies greater independence of *WS* from gravity, while a ratio further from zero suggests more gravitational influence on walking (Supplementary Figure S2). Equation (6) includes a multiplier of 100 to avoid extremely small numbers – according to equation (5), a free body will reach a velocity value of 102 m/s at 60 s, while walking speeds usually did not exceed 2 m/s.

### Linearly Weighted Summation

To estimate the weight of visual and physical body-based cues, we incorporated a linear weighted summation. Given that locomotion relies on multiple sensory cues, we can model it as weighted linear average to describe distinct features contributing to locomotion. In this regard, we define *WS* as a behavior integrating weighted contributions of visual cues and body-based cues (e.g., proprioceptive, vestibular) (Campos et al., 2014). The general model is as follows:

(7) $$W\,S=w_{v\,i\,s\,u\,a\,l\,c\,u\,e\,s}\,W\,S_{v\,i\,s\,i\,o\,n.d\,r\,i\,v\,e\,n}+w_{b\,o\,d\,y\,c\,u\,e\,s}\,W\,S_{b\,o\,d\,y.d\,r\,i\,v\,e\,n}$$

The variable *w* denotes the weight of the unimodal cue (i.e., visual or body-based). To estimate visual and body-based sensory weights, we assume that *WS* in conditions T_L_V_U_ (*WS*_*vis,up*_) and T_L_V_D_ (*WS*_*vis,down*_) is driven solely by uphill and downhill visual cues, respectively. Conversely, we assume that *WS* in conditions T_U_V_L_ (*WS*_*body,up*_) and T_D_V_L_ (*WS*_*body,down*_) is driven solely by the corresponding body-based (physical) cues:

(8) $$W\,S_{u\,p}=w_{v\,i\,s,u\,p}\,W\,S_{v\,i\,s,u\,p}+w_{b\,o\,d\,y,u\,p}\,W\,S_{b\,o\,d\,y,u\,p},$$

(9) $$W\,S_{d\,o\,w\,n}=w_{v\,i\,s,d\,o\,w\,n}\,W\,S_{v\,i\,s,d\,o\,w\,n}+w_{b\,o\,d\,y,d\,o\,w\,n}\,W\,S_{b\,o\,d\,y,d\,o\,w\,n}$$

Here, *WS*_*up*_represents congruent uphill walking (T_U_V_U_) and *WS*_*down*_ congruent downhill walking (T_D_V_D_). The calculation of *w* for visual and body-based cues was according to the following equations:

(10) $$w_{v\,i\,s\,u\,a\,l}=\frac{W\,S_{c\,o\,m\,b\,i\,n\,e\,d}-W\,S_{b\,o\,d\,y}}{W\,S_{v\,i\,s\,i\,o\,n}-W\,S_{b\,o\,d\,y}},w_{b\,o\,d\,y}=\frac{W\,S_{c\,o\,m\,b\,i\,n\,e\,d}-W\,S_{v\,i\,s\,i\,o\,n}}{W\,S_{b\,o\,d\,y}-W\,S_{v\,i\,s\,i\,o\,n}}$$

Finally, equations (7–10) imply that *w_visual_* + *w_body_* = 1. Predictions of walking speed relied on equations (8) and (9). However, to avoid repetition of mathematical factors in the equations, the predicted behavior of uphill walking and downhill walking was estimated using the opposite unimodal weights (i.e., *predicted uphill walking* was calculated using *w_visual_* and *w_body_* derived from downhill conditions, whereas *predicted downhill walking* was based on weights derived from uphill conditions). Our use of opposite unimodal weights (i.e., downhill weights for uphill walking, and uphill weights for downhill walking) is consistent with the conceptual hypothesis that sensory weighting ratios inherent to the individual dictate WS in uphill- and downhill-related conditions.

### Calculation of Joint Angles and Spatiotemporal Gait Parameters

In addition to WS, we evaluated gravity- and vision-based effects on joint angles and spatiotemporal gait parameters. We calculated joint angles (i.e., from hip, knee, ankle, elbow, shoulder girdle, pelvic frontal, trunk sagittal and trunk frontal) and spatiotemporal gait parameters (i.e., step length, stride length, stance time and cadence). Detailed definitions of the gait parameters have been previously published (Kimel-Naor et al., 2017). Briefly, the pelvic frontal tilt and the shoulder girdle rotation were calculated in relation to the global medio-lateral axis, while the trunk angles in the sagittal and frontal planes were calculated in relation to the vertical axis. The remaining joint angles were calculated based on local angles (i.e., defined by three markers and corresponding two vectors). For congruent conditions (T_U_V_U_, T_L_V_L_, and T_D_V_D_), aiming to extract data from steady locomotion, the calculation was in the 60s post-transition window, specifically over the last 12-s period of *WS* with a coefficient of variance less than 2% – an approach similar to the calculation of ssv (most often from the last 12 s; i.e., from 48 to 60 s post-transition). For incongruent conditions (T_U_V_L_, T_U_V_D_, T_L_V_U_, T_L_V_D_, T_D_V_U_, and T_D_V_L_), aiming to identify adjustments driven by the incongruent sensory input, we calculated joint angles from the first 20 s post-transition. In all cases, to characterize postural adjustments, we extracted the averaged minimum, the averaged maximum and the range of motion (i.e., maximum displacement) of joint angles from six homogeneous (i.e., free of noise or movement artifacts) and consecutive gait cycles. For comparison purposes, we used Marginalization (see below).

### Marginalization

We define marginalization as the process of identifying a characteristic (e.g., treadmill inclination) of the nine experimental conditions to serve as reference for cross-condition analyses. In this regard, we *marginalize* conditions according to treadmill inclination (i.e., up, level or down) to quantify deviations from the assumption that conditions with the same treadmill inclination promote similar gait behaviors and lead to comparable outcomes. Accordingly, we assume that any differences in outcomes among these conditions (with the same treadmill inclination) are attributable to the distinct visual input. Therefore, there were three types of comparisons. For treadmill uphill, we compared outcomes among conditions T_U_V_U_, T_U_V_L_, and T_U_V_D_. The corresponding comparisons were made for treadmill level (conditions T_L_V_U_, T_L_V_L_, and T_L_V_D_) and for treadmill downhill (conditions T_D_V_U_, T_D_V_L_, and T_D_V_D_).

### Calculation of the Coefficient of Variance of Walking Speed

The coefficient refers to walking variability and serves to estimate the extent of gait alterations following environmental transitions in the nine experimental conditions. We computed the coefficient of variance for the raw (non-normalized) *WS* values:

(11) $$C\,o\,e\,f\,f\,i\,c\,i\,e\,n\,t\,o\,f\,v\,a\,r\,i\,a\,n\,c\,e_{W\,S}=\frac{S\,t\,a\,n\,d\,a\,r\,d\,D\,e\,v\,i\,a\,t\,i\,o\,n_{W\,S}}{A\,v\,e\,r\,a\,g\,e_{W\,S}}*100\%$$

For comparison purposes, we applied Marginalization.

### Statistical Analyses

Values for outcomes are expressed as mean ± 1 SD unless otherwise stated. Walking speed was normalized and expressed as percentage of ssv (Equation 1). Spearman’s test was used to compute correlations between gravito-inertial effects (braking effect vs. exertion effect), vision-induced changes in the coefficient of variance of walking speed, as well as spatiotemporal gait parameters for congruent uphill and downhill walking conditions. The correlational analysis of gravito-inertial effects included separate time windows covering the entire experimental period. Paired *t*-tests (two-tailed, *P* < 0.05) were used for within-participant comparison of continuous outcomes (e.g., walking speed, postural adjustments, coefficient of variance of walking speed) for pairs of conditions (e.g., T_U_V_U_ vs. T_D_V_D_) and detection of visually induced effects (e.g., T_L_V_U_ vs. T_L_V_L_). *P*-values in the description of changes in walking speed in the nine experimental conditions refer to the comparison between averaged walking speed of the effect (20 s after transition) vs. ssv in each condition.

## Results

We assume that congruent conditions represent real-life situations: treadmill level – vision level (T_L_V_L_), treadmill up – vision up (T_U_V_U_) and treadmill down – vision down (T_D_V_D_) (Figure 1B). To evaluate our hypotheses regarding the effect of incongruence on walking adaptation, we first characterized the effect on walking speed of a congruent transition from level walking. Walking speed remained largely constant during level walking (*P* = 0.471 in the comparison of walking speed 20 after –no- transition with steady-state velocity: ssv) (Figure 2, middle panel). In contrast, the transition from level to uphill walking (Figure 2, upper-left panel) led to a rapid decrease in walking speed of −33 ± 15%_*ssv*_ (an average reduction at the peak of 33% compared to ssv) in *all participants*, beginning at 3.85 ± 1.10 s post-transition (i.e., after transition starts) (*P* < 0.001, walking speed 20 s after transition vs. ssv). Following the initial rapid decrease, walking speed remained relatively unchanged for the duration of the trial (range: [−18.7%_*ssv*_, −21.4%_*ssv*_]). Conversely, transition to downhill walking (Figure 2, lower-right panel) was characterized by an initial period of no change, followed by a comparatively *delayed* increase in walking speed beginning at 6.47 ± 3.63 s post-transition, with an average increase of 13% compared with ssv (+13 ± 23%_*ssv*_) (*P* = 0.071). Notably, although walking speed for the group increased, walking speed for three participants actually decreased with transition to downhill walking.

Visual cues proved to be a key determinant in walking adaptations. First, transition from congruent level walking to an incongruent inclination of the visual scene altered walking. For condition T_L_V_D_, in which the treadmill remains level but there is a downhill visual transition, walking speed decreased and then returned to ssv; maximal decrease was at 7.65 ± 1.99 s to −21 ± 16%_*ssv*_ (*P* = 0.024), with a decrease evident in fourteen of the fifteen individual participants (Figure 2, middle-right panel). Conversely, for condition T_L_V_U_, in which the treadmill remains level but there is an uphill visual transition, walking speed increased and then returned to treadmill level walking speed; maximal increase was at 12.4 ± 4.50 s to +16 ± 11%_*ssv*_ (*P* = 0.003), with an increase evident in 11 of 15 participants (Figure 2, middle-left panel). Critically, in these conditions, only visual cues changed, with body-based (physical) cues remaining constant.

In conditions with a transition from congruent level walking to incongruent uphill inclination of the treadmill, walking speed decreased as in the congruent transition to uphill walking, but the decrease was more pronounced (Figure 2, upper row). In condition T_U_V_L_ (visual scene remains level), walking speed dropped to −42% ± 25_*ssv*_ at 9.59 ± 3.98 s (*P* < 0.001), and in condition T_U_V_D_ (visual scene transitions downhill), walking speed dipped to −62 ± 25%_*ssv*_ at 7.97 ± 1.75 s (*P* < 0.001). The pattern was found in all 15 participants. In both incongruent conditions, walking speed gradually stabilized at a comparable velocity to congruent uphill walking. Conversely, in conditions transitioning to incongruent downhill inclination of the treadmill, walking speed increased similarly to the congruent transition to downhill walking (Figure 2, lower row). In condition T_D_V_L_ (visual scene remains level), walking speed rose to +15 ± 21%_*ssv*_ at 11.1 ± 5.66 s (*P* = 0.045), with an increase evident in 12 of 15 participants. In condition T_D_V_U_ (visual scene transitions uphill), walking speed climbed to +22 ± 17%_*ssv*_ at 11.6 ± 5.53 s (*P* = 0.012), with an increase in thirteen individual participants. In both of these incongruent conditions, walking speed progressively returned to a velocity comparable to congruent downhill walking speed. Remarkably, an incremental effect of visual incongruence seems evident, with the visual scene appearing to enhance the physical (treadmill) effect (Figure 2, rightmost column).

To estimate and characterize the role of gravity in the locomotor adaptations described above, we compared walking speed on inclined planes (i.e., congruent conditions T_U_V_U_ and T_D_V_D_) versus predicted (theoretical) deceleration/acceleration of a free body (i.e., with no frictional forces) moving on an identical (10°) plane uphill or downhill, respectively. We assume that the difference between change in human walking speed and change in the movement of a free body on an inclined trajectory represents human effort to counteract effects of gravity on walking adaptations (Figure 3A).

In downhill walking, gravity boosts walking speed. However, this gravitational boost is not the sole determinant of walking speed. If it were, the increase in walking speed would be equal to that of a body moving with no friction. Instead, a *braking effect* also acts upon downhill walking speed in an effort to reduce gravitational acceleration. Conversely, in uphill walking, gravity slows walking speed and an *exertion effect* is applied in an effort to counteract the slowing and maintain walking speed. We found high correlation between braking and exertion effects (Figure 3B, Spearman correlation: 0.6 < r_*s*_ < 0.7, *P* < 0.01).

**FIGURE 4:**
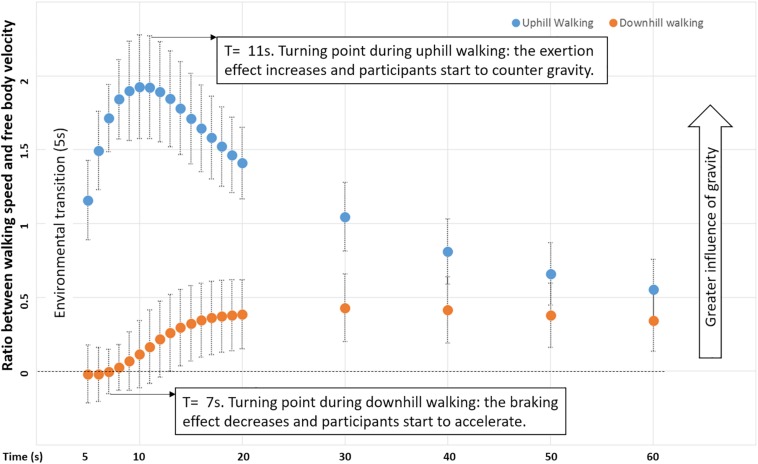
Ratio of gravity-induced behavior in uphill and downhill walking. The ratio between walking speed and velocity of a free-moving body over time for downhill and uphill congruent conditions (mean across participants; error bars represent standard error) (Supplementary Figure S2 and Section Materials and Methods). The *turning* (i.e., time) points at which participants start to reduce the braking effect and begin succumbing to gravitational acceleration in downhill walking (7 s) and, conversely, apply the exertion effect to counter gravitational deceleration in uphill walking (11 s) are indicated.

To compare changes in braking and exertion effects over time (post-transition), we computed a normalized ratio between areas under the curve: walking speed (in congruent downhill and uphill walking) divided by free body velocity (Figure 4). For both downhill and uphill walking, the higher the ratio (i.e., further from zero), the stronger the effect of gravity and the weaker the braking or exertion effect, respectively. This analysis revealed a robust differential response to gravity in uphill vs. downhill walking, until approximately 30 s post-transition (*P* < 0.01). During this period, uphill walking initially showed a larger ratio, reflecting a strong decelerating influence of natural gravity peaking at 11 s. The exertion effect then followed this peak, as participants expended effort to maintain walking speed. In contrast, the ratio was smaller for downhill walking, reflecting a weaker accelerating influence of natural gravity and a strong braking effect during the initial 7 s post-transition.

**FIGURE 5:**
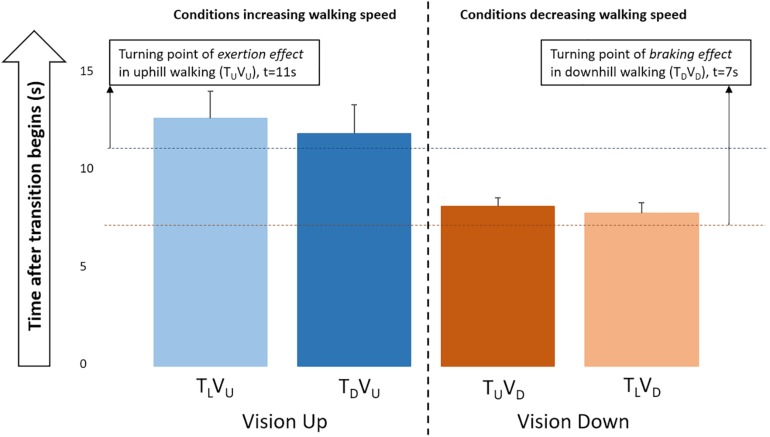
Temporary adaptation of walking speed during initial response. Time of maximal change in conditions increasing (left) and decreasing (right) walking speed in the first 20 s post-transition (see Figure 2, rightmost column). Error bars represent standard error. Dashed horizontal lines mark the *turning points* when participants begin countering gravitational deceleration (increase exertion) in congruent uphill walking (11 s) or begin yielding to gravitational acceleration (decrease braking) in congruent downhill walking (7 s). Thus, humans exercise braking to maintain walking stability with a downhill shift sooner than they apply exertion to attain stability with an uphill shift. Critically, this pattern was evident even in conditions of incongruence between visual and physical cues, suggesting that visual cues predominate over physical cues for perception of environmental changes.

Having characterized the relationships between gravity-induced and braking/exertion effects for walking adaptation in congruent conditions, a comparison with the incongruent conditions can now be made to examine the impact of visual cues. Interestingly, the initial acceleration in conditions T_L_V_U_ and T_D_V_U_ (incongruent uphill visual transitions) peaked at a time similar to that of the congruent uphill condition (∼11 s), and the deceleration in conditions T_U_V_D_ and T_L_V_D_ (incongruent downhill visual transitions) was largest at a time similar to that of the congruent downhill condition (∼7 s) (Figure 5).

**FIGURE 6:**
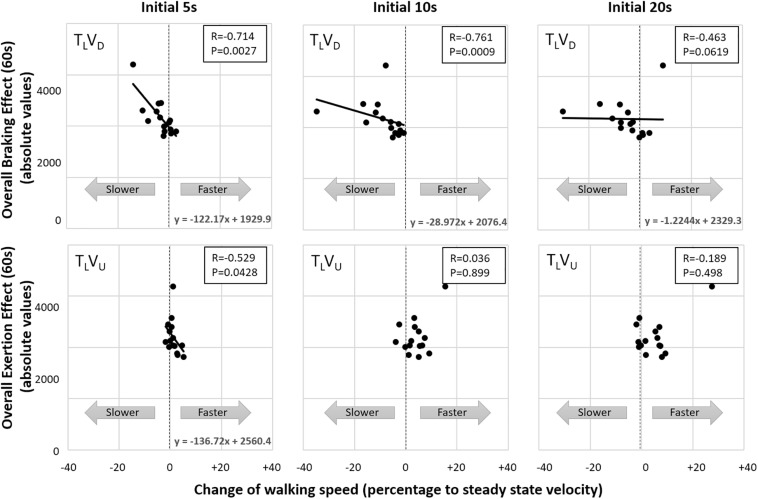
Correlation between early visual shifts and gravity-induced walking adaptation over entire 60-s post-transition period. Correlations (rs) show that vision-induced change in walking speed in the virtual downhill condition (T_L_V_D_) after 5 and 10 s post-transition are significantly correlated with the longer-term gravity-induced changes (characterized by the braking effect) in actual downhill walking (T_D_V_D_). In contrast, change in walking speed induced by uphill visual transition (T_L_V_U_) was correlated with the longer-term gravity-induced changes (characterized by the exertion effect) at only 5 s post-transition. Thus, visual shifts alone (indirectly) predict veridical gravity-induced walking adaptations, with prediction persisting longer for downhill shifts.

To better understand how early visual cues serve to indirectly predict walking adaptation effects over the entire course of the post-transition period, we computed correlations between gravity-induced walking adaptation over the 60-s post-transition period (from the congruent T_D_V_D_ downhill/braking and T_U_V_U_ uphill/exertion conditions, respectively) and changes in walking speed (i.e., acceleration or deceleration) for the first 5, 10, and 20 s of the incongruent conditions in which only the visual scene shifted down (T_L_V_D_) or up (T_L_V_U_). We found that vision-induced change in walking speed after 5 and 10 s in the visual downhill (T_L_V_D_) condition was significantly correlated with walking adaptation (characterized by the braking effect) over the entire 60-s post-transition period. In contrast, vision-induced change in walking speed after 5 s (but not 10 s) in the visual uphill (T_L_V_U_) condition was correlated with walking adaptation (characterized by the exertion effect) over the entire post-transition period (Figure 6). Thus, early visual cues predict longer-term gravity-induced braking and exertion effects, respectively. However, relative to 10 s for the braking effect, visual cues predict the exertion effect only up to 5 s.

Thus far, we have focused on how visual cues give rise to expectations of gravity-related consequences to modulate initial locomotor adaptations following upward and downward environmental transitions. Let us now evaluate what occurs later. The stabilization of walking in uphill and downhill congruent conditions seems to be after the turning points in which participants start, respectively, applying the exertion effect (11 s) or reducing the braking effect (7 s) (Figures 2, 4). In addition, in incongruent conditions, after peak times (in either increasing or decreasing walking speed), participants gradually appeared to reject the discordant visual feedback, and walking began to resemble gravity-based (i.e., treadmill) walking (Figures 2, 5). To estimate the diminishing contribution of visual as compared with other sensory inputs, we implemented a linearly weighted summation (Methods) (Campos et al., 2014). This simple model assumes that congruent walking integrates unimodal cues – visual and body-based (e.g., proprioceptive, vestibular). Thus, uphill walking is the result of relative contributions from “vision up” and “treadmill up,” and downhill walking is the result of contributions from both “vision down” and “treadmill down.” Note that the relative contribution represents the weight of each unimodal cue, and the model assumes that the sum of weighted unimodal cues is always equal to one. This model facilitates estimation of the sensory reweighting of vision. For example, a weight of vision near zero indicates that locomotion predominantly relies on body-based cues.

**FIGURE 7:**
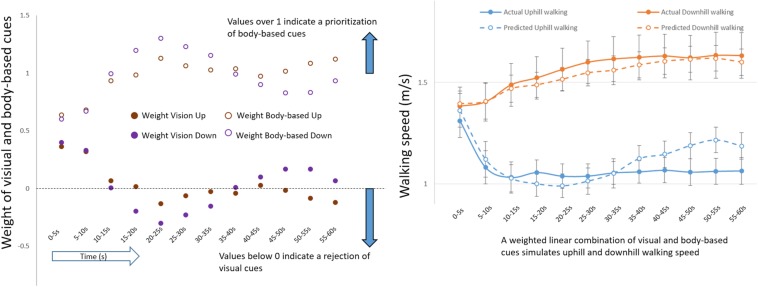
Sensory reweighting. **(Left)** Weight of visual (filled circles) and body-based (unfilled circles) cues during uphill and downhill (congruent) walking. **(Right)** Actual changes in congruent walking speed (solid lines) as predicted by model weights from incongruent conditions (dashed lines) (See the Linearly Weighted Summation section in Materials and Methods). Errors bars represent standard error.

Application of this model revealed that the relative weights of visual and body-based cues diverge following environmental transition, illustrating sensory reweighting (Figure 7, left panel). Gradually at post-transition, the weight of visual cues decreases over time, and the weight of body-based cues increases. Moreover, a linear combination of sensory weights derived from incongruent conditions was able to predict congruent walking (Figure 7, right panel).

**FIGURE 8:**
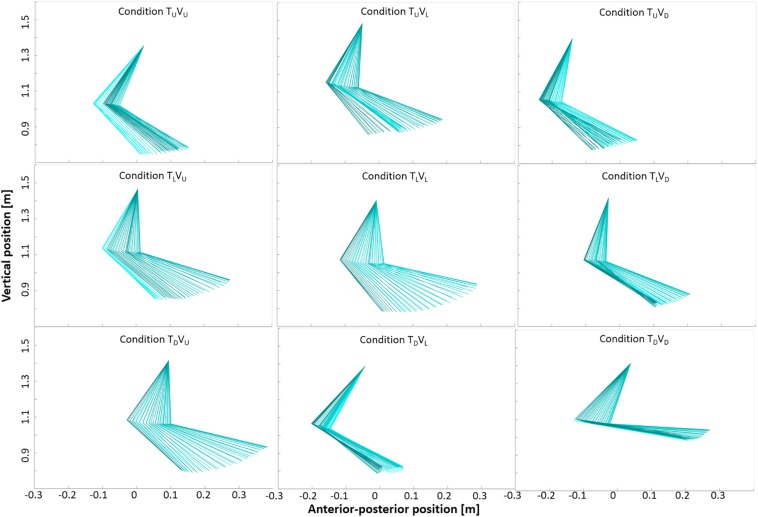
Arm and forearm movement in the nine experimental conditions. Sagittal plots of right upper-limb angles in one representative participant during a single gait cycle taken from 0 to 20 s post-transition (incongruent conditions) and from stable walking (congruent conditions: 48–60 s post-transition). Data from the shoulder, elbow and wrist defined the two segments of arm and forearm. See Table 1 for joint angles. Darker color indicates backward movement, and lighter color indicates forward movement. Visual cues appear to influence upper-limb movement during incongruent conditions in a manner consistent with the (visually) corresponding congruent condition. For example, in the middle row, incongruent conditions T_L_V_U_ and T_L_V_D_ approach the shape of congruent conditions T_U_V_U_ and T_D_V_D_, respectively.

**FIGURE 9:**
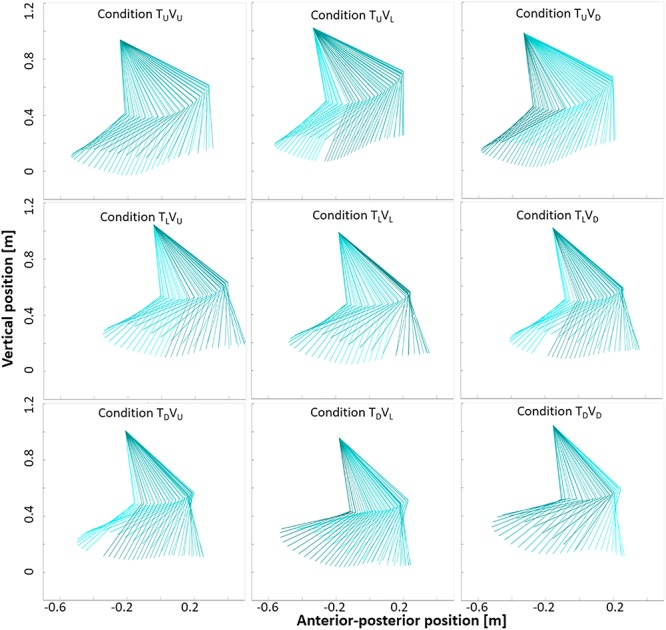
Thigh and shank movement in the nine experimental conditions. Sagittal plots of right lower-limb angles in one representative participant during a single gait cycle taken from 0 to 20 s post-transition (incongruent conditions) and from stable walking (congruent conditions: 48–60 s post-transition). Data from the pelvis, knee and ankle defined the two segments of thigh and shank. Darker color indicates backward movement, and lighter color indicates forward movement. Visual cues appear to influence lower-limb movement during incongruent conditions in a manner consistent with the (visually) corresponding congruent condition. In particular, range of movement appears consistently narrower in conditions with a downward visual transition (see Table 1).

To evaluate adaptations of postural adjustments, we first defined differences in joint angles for the congruent conditions. Our results were largely consistent with prior studies (Leroux et al., 2002; Dewolf et al., 2018). Uphill (relative to level) walking augmented range of motion of hip, shoulder girdle and trunk (frontal), while reducing that of knee and elbow – adjustments characteristic of deceleration (*P* < 0.01). In turn, downhill walking promoted broader range of motion of the knee and pelvis (sagittal and girdle), while reducing that of ankle and shoulder girdle – adjustments consistent with acceleration (*P* < 0.01) (Table 1 and Figures 8, 9). Although there were no differences in the range of motion of the trunk in the sagittal plane, both minimum and maximum angles were significantly smaller for downhill as compared to uphill walking. This reflects a forward tilt of the body during uphill walking and a backward tilt during downhill walking (Leroux et al., 2002; Dewolf et al., 2018).

**TABLE 1:** Postural adjustments.

		Treadmill level			Treadmill downhill			Treadmill uphill		
		Vision level	Vision uphill	Vision downhill	Vision level	Vision uphill	Vision downhill	Vision level	Vision uphill	Vision downhill
				
Joint	Condition	T_L_V_L_	T_L_V_U_	T_L_V_D_	T_D_V_L_	T_D_V_U_	T_D_V_D_	T_U_V_L_	T_U_V_U_	T_U_V_D_
	Parameter									
Trunk Frontal [°]	Range	5.30 ± 1.21	5.66 ± 1.39	6.06 ± 0.99^∗^	4.60 ± 1.61	4.56 ± 1.59	4.43 ± 1.63	9.23 ± 3.18	7.96 ± 3.27	8.92 ± 4.20
	Maximum	1.44 ± 3.01	2.22 ± 1.96	1.71 ± 3.31	2.04 ± 3.24	1.41 ± 3.89	1.45 ± 2.85	3.61 ± 3.54	2.84 ± 5.05	3.10 ± 4.52
	Minimum	−3.86 ± 3.13	−3.44 ± 2.88	−4.35 ± 2.34	−2.56 ± 2.77	−3.15 ± 3.87	−2.98 ± 3.22	−5.61 ± 4.97	−5.12 ± 3.80	−5.82 ± 5.17
Trunk Sagittal [°]	Range	4.58 ± 1.23	4.46 ± 1.05	4.34 ± 1.09	4.22 ± 1.03	4.19 ± 1.33	4.77 ± 1.72	5.79 ± 2.65	7.03 ± 5.91	6.22 ± 2.73
	Maximum	16.08 ± 2.54	16.65 ± 2.95^∗^	15.64 ± 2.68^+^	14.22 ± 4.87	15.19 ± 5.49	14.25 ± 4.10	20.83 ± 5.49	24.20 ± 7.82^∗^	19.55 ± 4.21^+^
	Minimum	11.51 ± 2.68	12.19 ± 2.98	11.3 ± 2.71^+^	10.00 ± 4.75	11.00 ± 5.18^∗^	9.47 ± 4.04^+^	15.04 ± 4.93	17.17 ± 4.48^∗^	13.32 ± 4.57^* +^
Pelvic Frontal [°]	Range	16.70 ± 4.24	16.59 ± 3.88	15.73 ± 2.92	18.24 ± 5.26	18.13 ± 5.38	18.33 ± 5.23	14.44 ± 4.09	14.81 ± 4.07	12.06 ± 4.32^∗^^+^
	Maximum	8.65 ± 5.49	8.25 ± 6.00	8.21 ± 5.63	7.73 ± 6.16	8.38 ± 4.95	8.36 ± 5.36	5.64 ± 4.65	6.01 ± 4.47	4.46 ± 5.06^+^
	Minimum	−8.05 ± 6.54	−8.33 ± 5.83	−7.52 ± 6.05^+^	−10.51 ± 6.18	−9.75 ± 6.13	−9.97 ± 6.89	−8.79 ± 5.15	−8.80 ± 5.66	−7.60 ± 4.93^* +^
Hip [°]	Range	43.87 ± 5.56	44.56 ± 6.83	41.43 ± 7.61	35.64 ± 9.63	35.37 ± 11.14	38.84 ± 12.22	55.68 ± 9.21	57.92 ± 8.09	47.85 ± 11.21^∗^^+^
	Maximum	33.95 ± 5.62	34.29 ± 5.26	33.97 ± 8.69	25.82 ± 4.05	26.35 ± 4.03	27.12 ± 4.10	52.91 ± 6.27	56.34 ± 7.81^∗^	46.56 ± 8.17^* +^
	Minimum	−9.92 ± 4.82	−10.27 ± 5.43	−7.46 ± 4.19^* +^	−9.82 ± 9.28	−9.02 ± 12.12	−11.72 ± 11.57	−2.77 ± 7.08	−1.58 ± 5.06	−1.29 ± 7.06
Ankle [°]	Range	39.98 ± 4.16	39.18 ± 5.82	37.46 ± 5.83^∗^	33.35 ± 5.68	33.35 ± 5.80	33.17 ± 5.14	42.04 ± 8.54	42.7 ± 5.81	32.99 ± 11.43^* +^
	Maximum	26.49 ± 6.40	26.55 ± 6.52	28.11 ± 6.61^* +^	22.85 ± 7.14	21.56 ± 6.89	21.33 ± 7.28	45.60 ± 5.51	43.9 ± 5.23^∗^	46.11 ± 5.39^+^
	Minimum	−13.50 ± 6.03	−12.6 ± 8.33	−9.35 ± 7.98^* +^	−10.5 ± 7.95	−11.8 ± 8.97	−11.8 ± 8.10	3.56 ± 9.42	1.19 ± 7.19	13.11 ± 12.46^* +^
Knee [°]	Range	63.99 ± 4.91	62.95 ± 5.07	60.29 ± 7.16^∗^	68.61 ± 4.03	68.54 ± 5.23	70.58 ± 3.94^* +^	53.41 ± 5.38	53.46 ± 3.55	50.61 ± 7.43
	Maximum	57.99 ± 4.50	57.95 ± 4.65	55.29 ± 9.41	63.90 ± 6.02	64.62 ± 5.63	64.65 ± 5.45	56.1 ± 4.31	55.05 ± 3.90	52.88 ± 6.19^∗^
	Minimum	−6.00 ± 3.42	−5.00 ± 3.59^∗^	−5.00 ± 5.29	−4.71 ± 3.24	−3.92 ± 4.08	−5.93 ± 3.71^+^	2.69 ± 5.41	1.59 ± 4.57	2.27 ± 4.70
Shoulder Girdle [°]	Range	11.38 ± 3.23	11.51 ± 3.22	13.43 ± 3.81^* +^	8.66 ± 2.18	9.61 ± 3.09	8.82 ± 2.55	17.60 ± 6.25	16.10 ± 5.56	16.87 ± 5.91
	Maximum	5.69 ± 2.53	5.24 ± 2.59	6.18 ± 2.65	2.9 ± 3.06	3.69 ± 2.96^∗^	3.47 ± 3.12	7.51 ± 4.53	6.72 ± 3.86	7.25 ± 3.93
	Minimum	−5.69 ± 3.12	−6.27 ± 2.94	−7.25 ± 2.80^* +^	−5.76 ± 3.97	−5.93 ± 3.76	−5.36 ± 3.76	−10.09 ± 3.42	−9.38 ± 3.96	−9.62 ± 5.02
Elbow [°]	Range	32.81 ± 13.16	31.22 ± 11.75	27.21 ± 10.47^* +^	38.70 ± 16.62	38.74 ± 13.87	39.28 ± 13.92	19.49 ± 6.57	20.7 ± 9.25	18.64 ± 9.56
	Maximum	178.89 ± 7.02	180.87 ± 8.14	178.48 ± 6.61	176.68 ± 7.45	173.86 ± 7.06	172.72 ± 14.88	173.01 ± 6.94	174.06 ± 6.83	172.3 ± 10.04
	Minimum	146.06 ± 9.37	149.65 ± 10.98	151.27 ± 7.15^∗^	137.98 ± 12.12	135.12 ± 14.91	133.44 ± 12.91	153.52 ± 7.31	153.35 ± 6.68	153.66 ± 13.56

*Effects of physical (treadmill) and virtual (visual scene) inclinations on joint angles. Values appear as mean ± 1 SD. ^∗^*P* < 0.05 in comparison with vision level. ^+^*P* < 0.05 in comparison with vision uphill. Only significant comparisons between conditions with the same treadmill inclination are shown (e.g., right panel: T_*U*_V_*L*_, T_*U*_V_*U*_ and T_*U*_V_*D*_; see Marginalization above).*

Overall, for the incongruent conditions, incongruent downhill transition of the visual scene led to smaller trunk (sagittal) angles (i.e., backward body tilt, consistent with braking effect) relative to incongruent uphill transitions of the visual scene (i.e., forward body tilt, consistent with exertion effect) (*P* < 0.05). In incongruent conditions with level treadmill, range of motion during uphill transition of the visual scene (condition T_L_V_U_) did not significantly differ from level walking, while downhill transition of the visual scene (condition T_L_V_D_) triggered adjustments of knee, shoulder and elbow similar to congruent uphill walking (deceleration). Furthermore, while an incongruent uphill transition of the visual scene with downhill treadmill (T_D_V_U_) reduced the overall range of motion of the knee (compared to congruent downhill walking), an incongruent downhill transition of the visual scene with uphill treadmill (T_U_V_D_) narrowed the range of motion of the pelvis frontal, hip and ankle (in comparison with congruent uphill walking) (Table 1). In summary, mere transitions of visual scenes led to postural adjustments consistent with braking and exertion effects, most prominently in conditions inducing *deceleration* (rather than *acceleration*) of walking speed.

**FIGURE 10:**
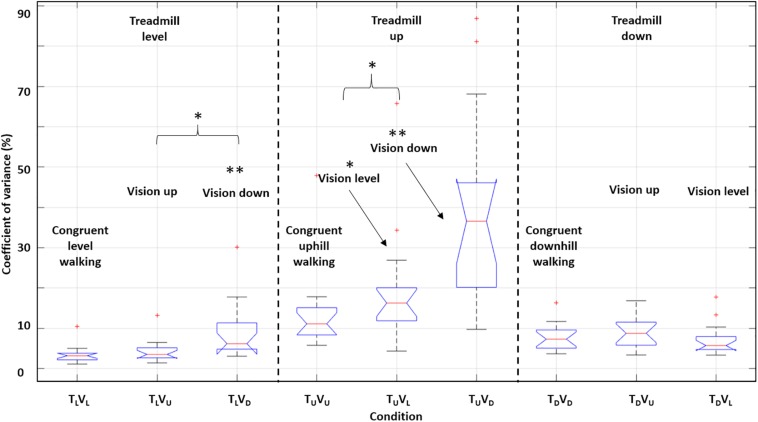
Impact on the coefficient of variance of walking speed induced by incongruent sensory information. Boxplots depicting coefficient of variance of walking speed during the first 20 s post-transition. For each boxplot, the central red line indicates the median, and the upper and lower edges indicate interquartile range. Outliers are plotted using red ‘+’ symbols. Here we marginalized according to the treadmill inclination. Vision down led to more variable gait in conditions with treadmill level and up. There were no differences across conditions with treadmill down. For each treadmill inclination, paired *t*-tests (two-tailed) were run between each incongruent condition and the congruent condition, and between the two incongruent conditions. ^∗^*P* < 0.05, ^∗∗^*P* < 0.01. Asterisks that appear without a brace indicate comparison to the corresponding congruent condition.

**FIGURE 11:**
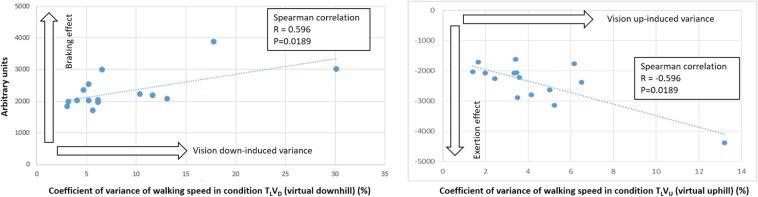
Vision-induced walking speed variance correlates with gravity-induced effects. Walking speed variance evident in visually induced incongruent conditions was significantly correlated with gravity-induced braking and exertion effects on walking speed in congruent conditions. Coefficient of variance of walking speed was calculated during the first 20 s post-transition. The greater the walking speed variance caused by an incongruent visual transition to a downhill scene (condition T_L_V_D_), the larger the braking effect. Likewise, the greater the walking speed variance caused by an incongruent visual transition to an uphill scene (condition T_L_V_U_), the larger the exertion effect. Note that the Spearman correlation was identical in both uphill and downhill cases, suggesting that the relationship between walking speed variance and gravity-induced changes in walking was the same for participants in both the downhill and uphill cases. These results support the presence of individualized susceptibility profiles for external vision-induced forces impinging upon locomotion.

To estimate adaptations in walking speed, we calculated the coefficient of variance of walking speed (Methods). Incongruent downhill visual transitions increased walking variance during level and uphill treadmill conditions; however, uphill visual incongruence did not significantly affect the coefficient of variance of walking speed (Figure 10). Thus, walking speed variance was more affected by *deceleration* (rather than *acceleration*) of walking speed. Notably, a correlational analysis revealed that participants with the highest variance induced by incongruent transitions of the visual scenes had the strongest braking and exertion effects (Figure 11).

Spatiotemporal gait parameters were consistent with adaptations of walking speed (Table 2). For example, incongruent downhill transitions of the visual scene decreased step and stride length, and this was consistent with the decrease in walking speed in these conditions (T_L_V_D_ and T_U_V_D_). In turn, incongruent uphill transitions of the visual scene (conditions T_L_V_U_ and T_D_V_U_) led to larger cadence in gait (Table 2). Also of note is that stance time during congruent downhill and uphill walking correlated with braking and exertion effects (*r*_*s*_ = 0.626, *P* = 0.016; and *r*_*s*_ = −0.594, *P* = 0.041, respectively; Spearman correlations), respectively.

**TABLE 2:** Spatiotemporal gait parameters.

	Treadmill level			Treadmill downhill			Treadmill uphill		
Condition	Vision level	Vision uphill	Vision downhill	Vision level	Vision uphill	Vision downhill	Vision level	Vision uphill	Vision downhill
			
Parameter	T_L_V_L_	T_L_V_U_	T_L_V_D_	T_D_V_L_	T_D_V_U_	T_D_V_D_	T_U_V_L_	T_U_V_U_	T_U_V_D_
Step length [m]	0.77 ± 0.11	0.79 ± 0.13	0.69 ± 0.12^* +^	0.71 ± 0.16	0.73 ± 0.17	0.76 ± 0.16	0.63 ± 0.11	0.69 ± 0.09	0.51 ± 0.15^* +^
Stride length [m]	1.51 ± 0.20	1.53 ± 0.24	1.36 ± 0.24^* +^	1.42 ± 0.31	1.46 ± 0.32	1.49 ± 0.33	1.24 ± 0.22	1.33 ± 0.18	0.96 ± 0.31^* +^
Stance duration [%]	0.64 ± 0.09	0.65 ± 0.11	0.69 ± 0.13^∗^	0.61 ± 0.12	0.55 ± 0.07^∗^	0.57 ± 0.08	0.76 ± 0.11	0.74 ± 0.08	0.98 ± 0.19^* +^
Cadence [1/min]	51.53 ± 6.88	53.5 ± 7.32^∗^	52.6 ± 7.90	56.14 ± 9.44	59.92 ± 6.76^∗^	55.71 ± 7.30^+^	47.75 ± 7.05	43.75 ± 5.69^∗^	41.75 ± 6.94^∗^

*Values appear as mean ± 1 SD. ^∗^*P* < 0.05 in comparison with vision level. ^+^*P* < 0.05 in comparison with vision uphill. Only significant comparisons between conditions with the same treadmill inclination are shown (e.g., right panel: T_*U*_V_*L*_, T_*U*_V_*U*_ and T_*U*_V_*D*_; see Marginalization above).*

## Discussion

We show that humans perceive gravity-related forces merely on the basis of visual cues and adapt locomotion in a manner anticipating the corresponding gravity-based cues. This study provides evidence that a mechanism of (visually induced) indirect prediction modulates locomotion based on our perception of gravity, and a mechanism of sensory reweighting ultimately stabilizes locomotion following environmental changes. We identify and describe the concepts of braking and exertion effects: inherent locomotor adaptations in opposition to gravitational force during downhill and uphill walking, respectively. Our findings suggest that locomotor adaptations while walking on inclined planes predict gravitational effects in a highly specific manner. In congruent conditions, participants promptly activate the braking effect to counteract the gravitational boost associated with downhill walking, whereas the exertion effect occurs later (11 s) and leads participants to counter gravitational deceleration during uphill walking.

By using virtual inclination incongruent with actual physical inclination, we were able to examine the temporal dynamics of sensory reweighting in the integration of visual and body-based cues to regulate locomotion on inclined planes. Incongruent downhill visual transitions led participants to slow down (i.e., braking effect), and incongruent uphill visual transitions to speed up (i.e., exertion effect). The contribution of visual perception predominates initially (first 10 s), and thereafter, locomotor adaptations appear to rely mainly upon body-based cues. Although spatiotemporal gait parameters were generally consistent with changes in walking speed, participants mainly made postural adjustments concurrently with reducing walking speed; thus slowing of walking speed was associated with more variable gait.

### Interpretation of Findings and Comparison to the Literature

Both braking and exertion effects reflect volitional effort to oppose the natural tendencies imposed by gravity. The high correlation between these effects at multiple time points (Figure 3B) suggests similar counter-gravity effort in both uphill and downhill walking. The counter-gravity effort during locomotor adaptations may be attributable to and characterized by perceived gravitational force. Our results further suggest that perception of gravity seems to dictate the time course of locomotor adaptations in transitions from level walking to congruent and incongruent conditions.

The differential response between uphill and downhill walking measured by the ratio of gravity-induced behavior demonstrate an intriguing asymmetry, whereby participants evidenced greater susceptibility to the slowing effect of gravity when ascending, as compared with the effect of gravity to speed them up when descending. Put differently, walking stability is maintained in response to a downward shift by braking sooner than applying exertion in response to an upward shift.

The time course of walking speed adaptations in incongruent acceleration and deceleration conditions were respectively similar to that of congruent uphill (peaked at ∼11 s) and downhill (largest at ∼7 s) conditions. Our findings are consistent with those of other studies using projected visual stimuli during treadmill walking (Mohler et al., 2007; O’Connor and Donelan, 2012). For example, Mohler et al. (2007) reported that changes in walking speed associated with congruent and incongruent visual flow occur 10.4–11.8 s after onset of conditions increasing speed and 7.3–9.2 s after onset of conditions decreasing speed. Given that the initial visual cue-based responses in incongruent conditions coincide with the period in which participants oppose gravity-induced behaviors during braking and exertion effects, visual cues alone appear to drive the estimation of gravity. Incongruent uphill visual transitions led participants to anticipate the exertion effect and this prolonged the increase of walking speed, whereas incongruent downhill visual transitions induced an early braking effect that decreased walking speed in anticipation of expected gravitational acceleration.

Our results on early visual cues predicting longer-term gravity induced effects show that the prediction-based decelerating effect was longer than the accelerating effect. This finding is consistent with the intuitive observation that humans find it easier – and preferable – to slow down rather than speed up. It has been demonstrated empirically that humans prefer walking speeds that minimize the cost of locomotion (Farris and Sawicki, 2011).

Our finding of a gradual return to body-based cues locomotion is consistent with sensory reweighting and the fact that actual physical resistance of the treadmill at a given inclination imposes the ultimate constraint upon walking speed (Prokop et al., 1997). The observed sensory reweighting dynamics shows that from about 15-20s after the transition and on, the effect of vision remains near zero, sometimes dropping to negative values, suggesting that visual cues contribute mainly to the initial adaptation of gait following environmental transition. Afterwards, gait control relies more prominently on body-based cues, and visual input is dismissed. This observation is in agreement with a previous study suggesting that, following optic flow changes, a transition from visual to leg-proprioceptive driven locomotion takes place (Prokop et al., 1997). This sensory reweighting reveals that the strongest braking effect occurs primarily during the period of highest visual weight (initial 7 s), while the exertion effect seems to stem largely from locomotion patterns led by body-based cues (after 11 s). Additionally, the ability of the linear combination model (based on sensory weights derived from incongruent conditions) to predict congruent walking suggests that locomotion relies on a mechanism of sensory reweighting that regulates the integration of multiple sensory cues for adapting to environmental changes over time.

The observation that participants with highest walking speed variance in incongruent conditions had the strongest gravity-induced effects (Figure 11) suggests that the ability to maintain a consistent gait, and the susceptibility to gravitational forces, seem to vary across participants. Hence, some individuals may be more or less prone to destabilizing effects of gait changes depending upon their perception of gravity. Different strategies for achieving less variable gait may arise from the fact that sensory reweighting does not operate in a uniform manner in all healthy adults (Brady et al., 2012), and coping with gravity and/or visual incongruence consequently varies among them.

Finally, as changes in spatiotemporal parameters during incongruent visual transitions were consistent with other locomotor adaptations (e.g., walking speed), our findings suggest that distorting perception of gravity with incongruent sensory information affects spatiotemporal gait patterns as well.

### Neural Mechanisms

A putative neural model to account for our findings is shown in Supplementary Figure S3. The proposed speculative, high-level model (see Supplementary Material) seeks to contextualize the observed locomotor modulations within potential underlying neural mechanisms and propose testable predictions derived from the model. The predictive system is critically based on an internal model of gravity (IMG) that regulates locomotion in accord with estimates of physical laws of gravity. In humans, brain regions compute the predicted effects of gravity by combining and comparing multiple sensory cues with an IMG (Lacquaniti et al., 2014; Balestrucci et al., 2017). The model explores the idea that locomotor adaptations, as those observed in our study, arise from an error signal generated by information from the visual-vestibular network incongruent with IMG predictions (Balestrucci et al., 2017). This mismatch may trigger the indirect prediction mechanisms, in which visual information predominates for a rapid, initial prediction and adjustment of gait pattern (O’Connor and Donelan, 2012). Such a mechanism of gait adjustment would be in accordance with the sufficiency of online visual control of locomotion (Zhao and Warren, 2015). Indirect prediction may occur via spinal reflexes (optimizing energy consumption) or central pattern generators (Pearson, 2004; Snaterse et al., 2011).

### Potential Implications

Future research should focus on determining the neural mechanisms associated with the visual perception of gravity and modulation of locomotion observed in our study. Studies may evaluate our putative model by measuring changes in brain activity during walking using such techniques as mobile fNIRS and EEG (Park et al., 2018). Specifically, these imaging modalities should be applied to explore the neural basis for the indirect prediction and sensory reweighting mechanisms proposed here. Studies might investigate the experimental conditions that trigger/activate the putative neural mechanisms/pathways. For example, manipulation of degree of inclination may allow precise definition of the task parameters that lead to the observed effects. We posit that smaller inclinations (<10°) may be less likely to trigger indirect prediction, and larger inclinations (>10°) may generate a longer period of sensory reweighting. Further, a priming manipulation might be added to our paradigm to determine the impact of higher-order cognitive control on the perception and action processes described. This would evaluate whether cognitive awareness can override the effect of incongruent sensory information on resultant behavior. Additional studies may explore the role of eye movements and scanning during locomotion under conditions of environmental transition and changes in gravitational effects. Ultimately, a detailed understanding of the neural mechanisms guiding human perception and action during locomotion may lead to VR-based paradigms for enhancing safe, accurate mobility (e.g., reducing falls) in normal and diseased individuals.

Finally, our findings come with potential implications within the area of VR-based rehabilitation of gait. For instance, to improve walking speed and gait parameters in patients with neurological conditions commonly associated to impaired gait, such as post-stroke patients and patients with Parkinson’s disease (Cano Porras et al., 2019). Researchers and clinicians might incorporate our paradigm of virtual inclinations in order to modulate aspects of gait according to theories of motor learning and neuroplasticity, based on the specific needs of each patient (Cano Porras et al., 2018). Additionally, the virtual inclinations paradigm may be useful for the assessment of visual dependency during human interaction within VR environments.

## Data Availability Statement

The datasets generated for this study are available on request to the corresponding author.

## Ethics Statement

The Institutional Review Board for Ethics in Human Studies at the Sheba Medical Center, Israel, approved the experimental protocol, and all participants gave written informed consent before being enrolled in the study.

## Author Contributions

MP conceived the idea. MP and DCP planned and carried out the experiments. DCP is the primary author, processed the experimental data. DCP, MP, and GD iteratively revised the manuscript. YB developed the virtual environments and participated in the data acquisition. RI and GZ supervised the study. All authors discussed the results and critically reviewed the manuscript.

## Conflict of Interest

GD is an employee of NeuroTrax Corporation. The remaining authors declare that the research was conducted in the absence of any commercial or financial relationships that could be construed as a potential conflict of interest.

## Supplementary Material

FIGURE S1 Calculation of area under the curve (AUC). Representative data from individual participants. The same method for calculating AUC was used for free body velocity and walking speed in the other experimental conditions.

FIGURE S2 Calculation of the ratio of gravity-induced behavior. Walking speed is for a single representative participant post-transition from level to uphill walking (congruent T_U_V_U_ condition). Calculation of free body velocity follows equation (5). Note that the turning point at 11 s (in the inset ratio graph) corresponds to the time at which walking stops decelerating due to gravity, and stabilization of locomotion begins.

FIGURE S3 Schematic model of neural perception-action mechanisms underlying the observed effects. A neural model that putatively accounts for our findings of locomotor modulations following perceived gravitational changes while walking. The core of this predictive system is an internal model of gravity that regulates locomotion in accord with estimates of physical laws of gravity. The model accounts for the mechanisms of indirect prediction and sensory reweighting, and incorporates the braking and exertion effects characterizing locomotor adaptations in our study. Relevant brain regions are shown. The inset panel is a schematic representation of the lumbosacral maps of LCPGs involved in the activation of muscles that play critical roles during uphill and downhill walking (Ivanenko et al., 2006; Pickle et al., 2016). *Green lines* point to brain areas involved in cue integration and sensory reweighting, the *orange line* emerges from the region proposed to subserve an internal model of gravity, *blue lines* are pathways involved in indirect prediction, and *gold lines* indicate proposed mechanisms of locomotor regulation and action. Dashed lines represent connectivity between areas in the central nervous system. The Discussion section on neural mechanisms (and the Putative neural model section in the Supplementary Materials) describes the scientific rationale for this model. MLR, mesencephalic locomotor region; LCPGs, locomotion central pattern generators, dITS, dorsal part of the inferior temporal sulcus [homologous to macaque dorsal medial superior temporal area (Huk et al., 2002)]; aIPS, anterior part of the intraparietal sulcus [homologous to macaque ventral intraparietal area (Grefkes and Fink, 2005)].

TABLE S1 Steady-state velocity.

PRESENTATION S1 Putative neural model (mechanisms and predictions).
