# Supplementary figures and images for: Seeing Gravity: Gait Adaptations to Visual and Physical Inclines – A Virtual Reality Study
Source: Front Neurosci. 2020 Jan 24;13:1308. doi: 10.3389/fnins.2019.01308 (PMC6992711; doi:10.3389/fnins.2019.01308)

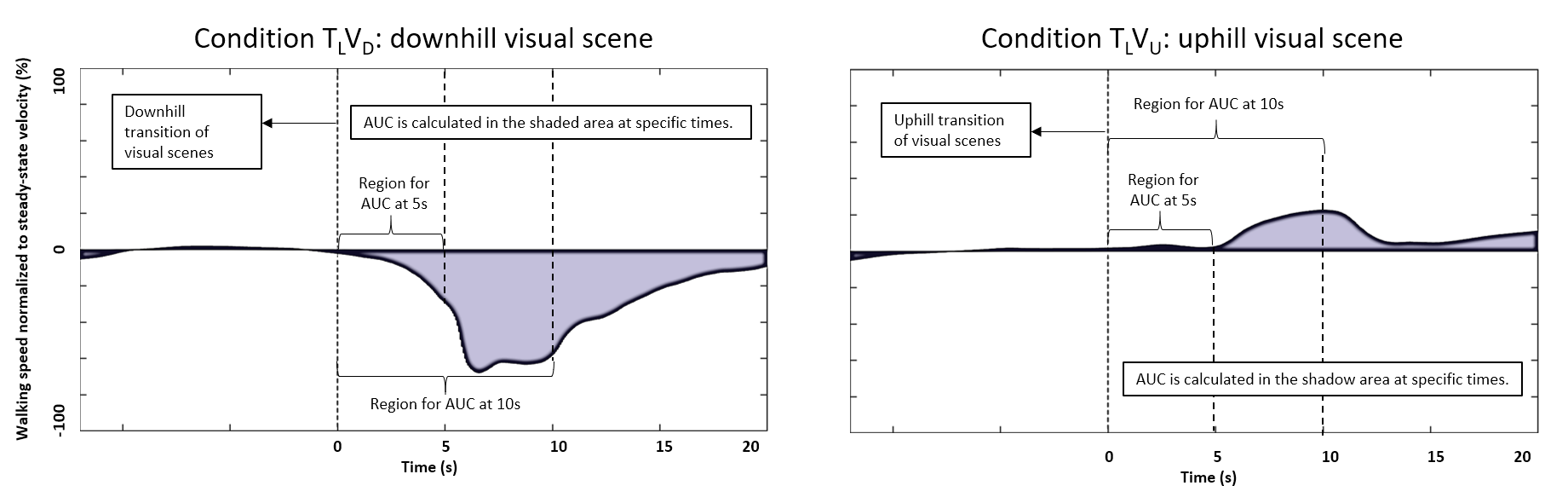

Supplement: FIGURE S1 — Calculation of area under the curve (AUC). Representative data from individual participants. The same method for calculating AUC was used for free body velocity and walking speed in the other experimental conditions. [file Image_1.tif]

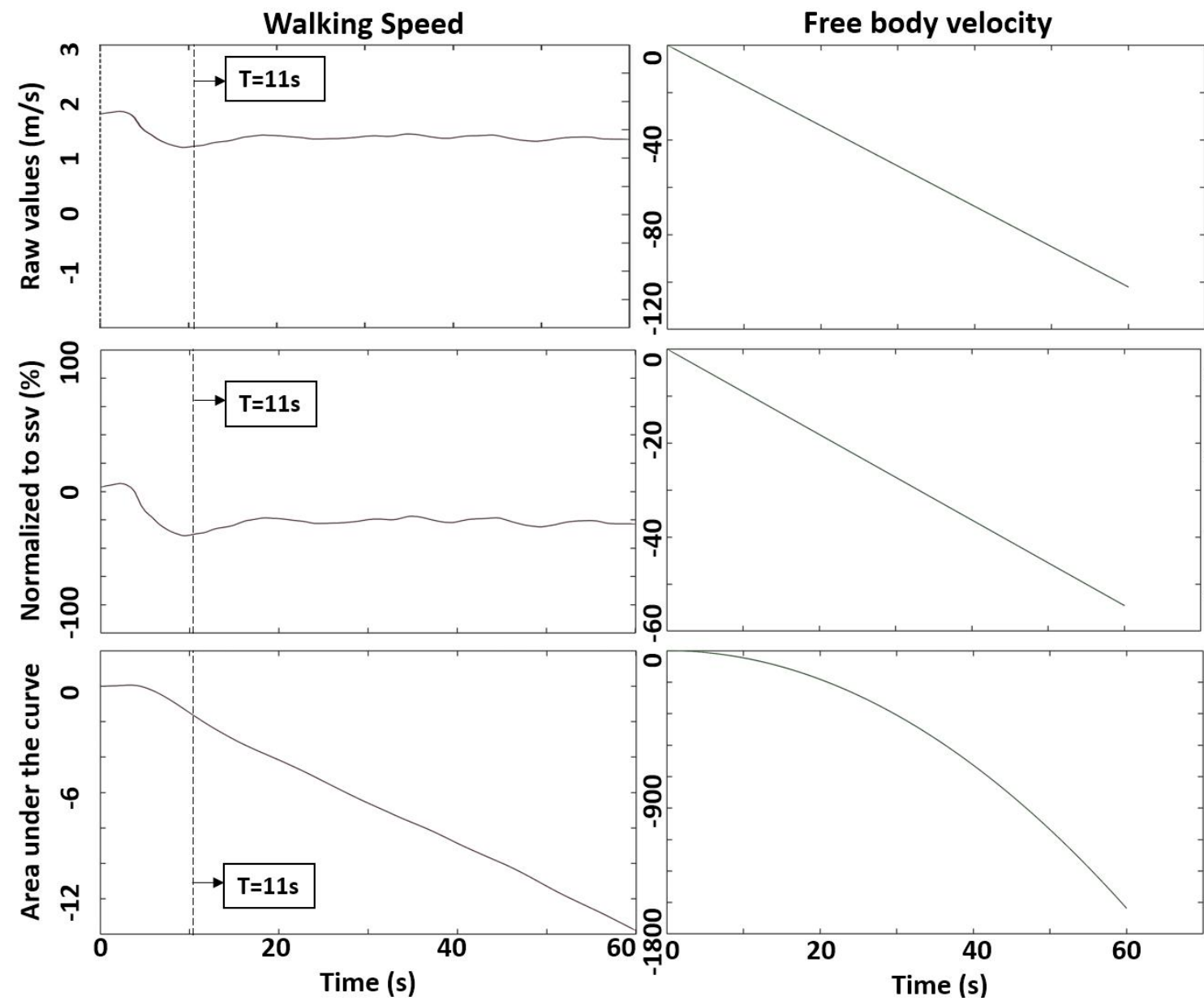

Ratio between areas under the curve

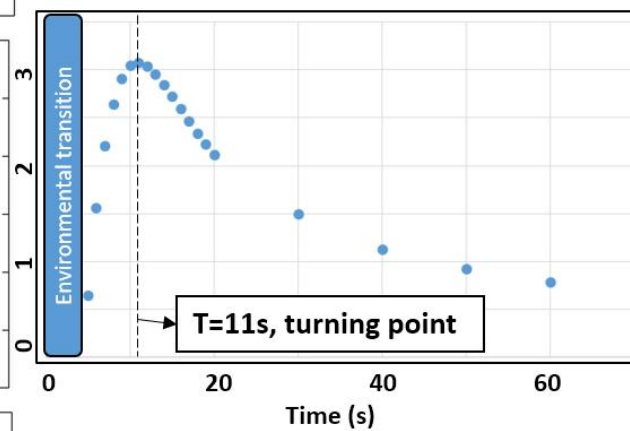

Supplement: FIGURE S2 — Calculation of the ratio of gravity-induced behavior. Walking speed is for a single representative participant post-transition from level to uphill walking (congruent TUVU condition). Calculation of free body velocity follows equation (5). Note that the turning point at 11 s (in the inset ratio graph) corresponds to the time at which walking stops decelerating due to gravity, and stabilization of locomotion begins. [file Image_2.pdf]

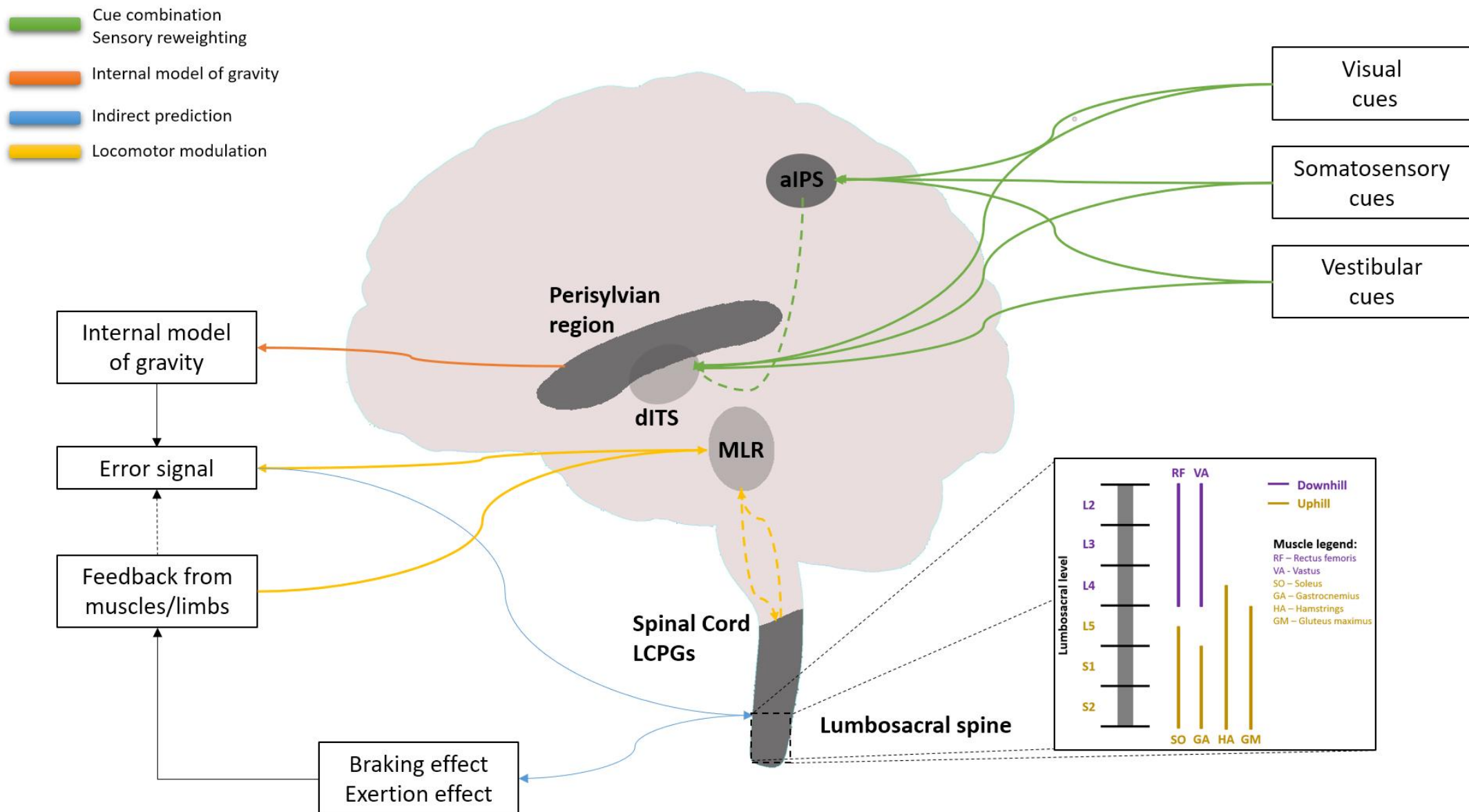

Supplement: FIGURE S3 — Schematic model of neural perception-action mechanisms underlying the observed effects. A neural model that putatively accounts for our findings of locomotor modulations following perceived gravitational changes while walking. The core of this predictive system is an internal model of gravity that regulates locomotion in accord with estimates of physical laws of gravity. The model accounts for the mechanisms of indirect prediction and sensory reweighting, and incorporates the braking and exertion effects characterizing locomotor adaptations in our study. Relevant brain regions are shown. The inset panel is a schematic representation of the lumbosacral maps of LCPGs involved in the activation of muscles that play critical roles during uphill and downhill walking (Ivanenko et al., 2006; Pickle et al., 2016). Green lines point to brain areas involved in cue integration and sensory reweighting, the orange line emerges from the region proposed to subserve an internal model of gravity, blue lines are pathways involved in indirect prediction, and gold lines indicate proposed mechanisms of locomotor regulation and action. Dashed lines represent connectivity between areas in the central nervous system. The Discussion section on neural mechanisms (and the Putative neural model section in the Supplementary Materials) describes the scientific rationale for this model. MLR, mesencephalic locomotor region; LCPGs, locomotion central pattern generators, dITS, dorsal part of the inferior temporal sulcus [homologous to macaque dorsal medial superior temporal area (Huk et al., 2002)]; aIPS, anterior part of the intraparietal sulcus [homologous to macaque ventral intraparietal area (Grefkes and Fink, 2005)]. [file Image_3.pdf]
