# Supplementary material for: Seeing Gravity: Gait Adaptations to Visual and Physical Inclines – A Virtual Reality Study
Source: Front Neurosci. 2020 Jan 24;13:1308. doi: 10.3389/fnins.2019.01308 (PMC6992711; doi:10.3389/fnins.2019.01308)
Supplement: TABLE S1 — Steady-state velocity. [file Table_1.docx]

**Table S1. Steady-state velocity.**

|  | **Treadmill Level** | | | **Treadmill Downhill** | | | **Treadmill Uphill** | | |
| --- | --- | --- | --- | --- | --- | --- | --- | --- | --- |
|  | **Vision Level** | **Vision**  **Uphill** | **Vision Downhill** | **Vision Level** | **Vision Uphill** | **Vision Downhill** | **Vision Level** | **Vision Uphill** | **Vision Downhill** |
| **Condition** | **T_L_V_L_** | **T_L_V_U_** | **T_L_V_D_** | **T_D_V_L_** | **T_D_V_U_** | **T_D_V_D_** | **T_U_V_L_** | **T_U_V_U_** | **T_U_V_D_** |
| **Walking speed [m/s]** | 1.40±0.31 | 1.40±0.37 | 1.40±0.33 | 1.38±0.38 | 1.42±0.33 | 1.38±0.27 | 1.40±0.29 | 1.35±0.32 | 1.39±0.31 |

Values shown as mean ± standard deviation.
