# Supplementary material for: Seeing Gravity: Gait Adaptations to Visual and Physical Inclines – A Virtual Reality Study
Source: Front Neurosci. 2020 Jan 24;13:1308. doi: 10.3389/fnins.2019.01308 (PMC6992711; doi:10.3389/fnins.2019.01308)
Supplement: PRESENTATION S1 — Putative neural model (mechanisms and predictions). [file Presentation_1.pdf]

Putative neural model (mechanisms and predictions)

Neural Mechanisms

There is evidence that mechanisms of cue integration and sensory reweighting govern human spatial navigation (Sun et al., 2004; Chen et al., 2017); for a review, see (Campos and Bülthoff, 2011). Though the neural basis for mechanisms of multisensory integration following environmental transitions during locomotion is yet to be elucidated in humans (Park et al., 2018), we can glean relevant insights from monkey studies.

In the macaque, the dorsal part of the medial superior temporal area (MSTd) (Gu et al., 2008) and the ventral intraparietal area (VIP) (Bremmer et al., 2002) (homologous to the dorsal side of the occipital continuation of the inferior temporal sulcus (Huk et al., 2002) and the anterior region of the intraparietal sulcus (Grefkes and Fink, 2005), respectively, in the human) have been shown to play a key role in both perceptual cue integration and multisensory analysis of self-motion (Bremmer et al., 2002; Gu et al., 2008; Chen et al., 2011; Fetsch et al., 2012). VIP and MSTd respond selectively to optic flow and regulate navigation (Duffy and Wurtz, 1991; 1995; Bremmer et al., 2002). Indeed, these areas appear to respond sequentially: vestibular processing of translational motion occurs first in VIP and then (within 100ms) in MSTd (Chen et al., 2011). It was suggested that similar multisensory integration processes occur in humans (Gu et al., 2008; Fetsch et al., 2009; Fetsch et al., 2012). Studies also suggest that the inferior temporal sulcus (Katsuyama et al., 2016) and the intraparietal sulcus (Bremmer et al., 2001; Grefkes and Fink, 2005; Regenbogen et al., 2018) are involved in multisensory integration and motion perception in humans.

Our findings related to anticipated gravitational effects suggest that the underlying neural circuitry is likely involved in our perception of gravity. In humans. brain regions compute

the predicted effects of gravity by combining and comparing multiple sensory cues with an internal model of gravity (IMG) (Lacquaniti et al., 2014; Balestrucci et al., 2017). The Sylvian fissure and surrounding (perisylvian) brain regions have been proposed to subserve the IMG; they are activated by multisensory cues and can modulate motor responses based on prediction of gravitational effects (Indovina et al., 2005; Maffei et al., 2015; Maffei et al., 2016; Balestrucci et al., 2017). The perisylvian region includes such receptors of gravitational information as the posterior insula and parietal operculum, with the parietal operculum contributing to detection of incongruities between the IMG and visual cues (Maffei et al., 2016). These two areas are part of the vestibular cortex, which plays a central role in spatial orientation, navigation and perception of gravity (Lopez and Blanke, 2011; Lopez et al., 2012). The vestibular cortex is also part of a multimodal visual-vestibular network that processes visual gravitational motion and includes subcortical (posterior thalamus, putamen, cerebellum and vestibular nuclei in the brainstem) brain regions (Lopez and Blanke, 2011; Lopez et al., 2012; Lacquaniti et al., 2013). The cerebellum plays a key role in the visual-vestibular network by relaying optic tract projections carrying retinal information derived from optic flow crucial for self-motion estimation (Lacquaniti et al., 2013). The cerebellum also contributes to our estimation of body orientation by processing afferent signals via dorsal spinocerebellar tracts (Bosco et al., 2006; Choi and Bastian, 2007) and influences locomotion via efferent projections to descending brainstem motor pathways (Orlovsky, 1972b; a; Choi and Bastian, 2007). Therefore, the IMG may incorporate physical estimates of gravitational mechanics [computed by the brainstem and cerebellum (Angelaki et al., 2004; Lacquaniti et al., 2013; Maffei et al., 2016)] into gravity-related expectations in the visual-vestibular network (Angelaki et al., 2004; Indovina et al., 2005; Lacquaniti et al., 2013; Maffei et al., 2016).

Presumably, locomotor adaptations in our study arise from an error signal generated by visual information incongruent with IMG predictions (Balestrucci et al., 2017) (**Figure S3**). This mismatch may trigger the indirect prediction mechanisms, in which visual information predominates for a rapid, initial prediction and adjustment of gait pattern (O'Connor and Donelan, 2012). Such a mechanism of gait adjustment would be in accordance with the sufficiency of online visual control of locomotion (Zhao and Warren, 2015). Indirect prediction may occur via spinal reflexes (optimizing energy consumption) or central pattern generators (Pearson, 2004; Snaterse et al., 2011). The synergic interactions among locomotion central pattern generators (LCPGs) are highly robust, time-adaptive and adjust to changing environmental contexts (Choi and Bastian, 2007). LCPGs regulate the activation of lumbosacral alpha motoneurons responsible for the activation of muscles with specialized functional roles in uphill and downhill walking (Ivanenko et al., 2006; Flash and Bizzi, 2016; Pickle et al., 2016). Therefore, LCPGs may be responsible for the synergic muscle activations that enable the braking and exertion effects in our study (**Figure S3**). Measuring spatiotemporal activation of such muscles according to spinal cord maps can be used to test the mechanism of indirect prediction and its associated braking and exertion effects. LCPGs respond to multisensory input and receive commands originating in motor cortex, brainstem and cerebellum before reaching the midbrain (mesencephalic) locomotor region (MLR). This region, in turn, receives afferent feedback from muscles and limbs (Prochazka and Ellaway, 2012). As functional human locomotion requires supraspinal input (Nielsen, 2003), this feedback loop may represent one key component for gradually correcting the error arising from sensory perceptual input inconsistent with the IMG. We hypothesize that feedback loop-driven gradual corrections reflect the sensory reweighting mechanism observed in our study (Campos et al., 2014; Assländer and Peterka, 2016).

Predictions of the model

Spinal cord maps chart the underlying pattern generators in specific spinal segments and their corresponding muscle innervation (Ivanenko et al., 2003; Ivanenko et al., 2006). Our model predicts that pattern generators controlling motoneuron activation in spinal segments innervating muscles involved in braking and exertion effects during (congruent) inclined walking will activate the identical motoneurons (and innervated muscles) in the presence of incongruent visual cues, thus inducing braking and exertion effects. More specifically, our model predicts that downward virtual inclines should activate the rectus femoris and vastus muscles involved in actual downhill walking/braking effect, and upward virtual inclines should activate the soleus, gastrocnemius, hamstrings, and gluteus maximus involved in actual uphill walking/exertion effect (Pickle et al., 2016). Following these effects of indirect prediction, we anticipate that with sensory reweighting, the muscles synergies will gradually return to their original state (i.e., prior to exposure to the virtual inclines) (**Figure 7**).

Dopamine pathways include descending projections that innervate the MLR and modulate locomotion via LCPGs in the spinal cord (Ryczko and Dubuc, 2017). Such neural mechanisms may be compromised in patients with Parkinson's disease (PD) (Sherman et al., 2015). As such, our model predicts that locomotor effects may be attenuated in PD patients; for example, onset of the braking/exertion effects may be delayed in PD, particularly when patients are "off" dopaminergic medication (Almeida et al., 2005; Chastan et al., 2009). Further, PD patients are better at performing actions involving lower energy cost (Gepshtein et al., 2014). We thus predict that PD patients would perform better when exposed to virtual inclinations with greater perceived gravitational boost (e.g., braking effect, downhill walking) and of lesser slope (e.g., 2° vs. 10° uphill).

Almeida, Q., Frank, J., Roy, E., Jenkins, M., Spaulding, S., Patla, A., et al. (2005). An evaluation of sensorimotor integration during locomotion toward a target in Parkinson's disease. *Neuroscience* 134(1), 283-293.

Angelaki, D.E., Shaikh, A.G., Green, A.M., and Dickman, J.D. (2004). Neurons compute internal models of the physical laws of motion. *Nature* 430(6999), 560.

Assländer, L., and Peterka, R.J. (2016). Sensory reweighting dynamics following removal and addition of visual and proprioceptive cues. *Journal of Neurophysiology* 116(2), 272-285.

Balestrucci, P., Daprati, E., Lacquaniti, F., and Maffei, V. (2017). Effects of visual motion consistent or inconsistent with gravity on postural sway. *Experimental Brain Research* 235(7), 1999-2010.

Bosco, G., Eian, J., and Poppele, R. (2006). Phase-specific sensory representations in spinocerebellar activity during stepping: evidence for a hybrid kinematic/kinetic framework. *Experimental Brain Research* 175(1), 83-96.

Bremmer, F., Klam, F., Duhamel, J.R., Hamed, S.B., and Graf, W. (2002). Visual–vestibular interactive responses in the macaque ventral intraparietal area (VIP). *European Journal of Neuroscience* 16(8), 1569-1586.

Bremmer, F., Schlack, A., Shah, N.J., Zafiris, O., Kubischik, M., Hoffmann, K.-P., et al. (2001). Polymodal motion processing in posterior parietal and premotor cortex: a human fMRI study strongly implies equivalencies between humans and monkeys. *Neuron* 29(1), 287-296.

Campos, J., and Bülthoff, H. (2011). Multisensory integration during self-motion in virtual reality. *Frontiers in the neural bases of multisensory processes. Taylor and Francis Group, London*, 603-627.

Campos, J.L., Butler, J.S., and Bülthoff, H.H. (2014). Contributions of visual and proprioceptive information to travelled distance estimation during changing sensory congruencies. *Experimental Brain Research* 232(10), 3277-3289.

Chastan, N., Do, M.C., Bonneville, F., Torny, F., Bloch, F., Westby, G.M., et al. (2009). Gait and balance disorders in Parkinson's disease: impaired active braking of the fall of centre of gravity. *Movement Disorders* 24(2), 188-195.

Chen, A., DeAngelis, G.C., and Angelaki, D.E. (2011). Representation of vestibular and visual cues to self-motion in ventral intraparietal cortex. *Journal of Neuroscience* 31(33), 12036-12052.

Chen, X., McNamara, T.P., Kelly, J.W., and Wolbers, T. (2017). Cue combination in human spatial navigation. *Cognitive Psychology* 95, 105-144.

Choi, J.T., and Bastian, A.J. (2007). Adaptation reveals independent control networks for human walking. *Nature Neuroscience* 10(8), 1055.

Duffy, C.J., and Wurtz, R.H. (1991). Sensitivity of MST neurons to optic flow stimuli. I. A continuum of response selectivity to large-field stimuli. *Journal of Neurophysiology* 65(6), 1329-1345.

Duffy, C.J., and Wurtz, R.H. (1995). Response of monkey MST neurons to optic flow stimuli with shifted centers of motion. *Journal of Neuroscience* 15(7), 5192-5208.

Fetsch, C.R., Pouget, A., DeAngelis, G.C., and Angelaki, D.E. (2012). Neural correlates of reliability-based cue weighting during multisensory integration. *Nature Neuroscience* 15(1), 146.

Fetsch, C.R., Turner, A.H., DeAngelis, G.C., and Angelaki, D.E. (2009). Dynamic reweighting of visual and vestibular cues during self-motion perception. *Journal of Neuroscience* 29(49)**,** 15601-15612.

Flash, T., and Bizzi, E. (2016). Cortical circuits and modules in movement generation: experiments and theories. *Current Opinion in Neurobiology* 41**,** 174-178.

Gepshtein, S., Li, X., Snider, J., Plank, M., Lee, D., and Poizner, H. (2014). Dopamine function and the efficiency of human movement. *Journal of cognitive neuroscience* 26(3)**,** 645-657.

Grefkes, C., and Fink, G.R. (2005). The functional organization of the intraparietal sulcus in humans and monkeys. *Journal of Anatomy* 207(1)**,** 3-17.

Gu, Y., Angelaki, D.E., and DeAngelis, G.C. (2008). Neural correlates of multisensory cue integration in macaque MSTd. *Nature Neuroscience* 11(10)**,** 1201.

Huk, A.C., Dougherty, R.F., and Heeger, D.J. (2002). Retinotopy and functional subdivision of human areas MT and MST. *Journal of Neuroscience* 22(16)**,** 7195-7205.

Indovina, I., Maffei, V., Bosco, G., Zago, M., Macaluso, E., and Lacquaniti, F. (2005). Representation of visual gravitational motion in the human vestibular cortex. *Science* 308(5720)**,** 416-419.

Ivanenko, Y.P., Grasso, R., Zago, M., Molinari, M., Scivoletto, G., Castellano, V., et al. (2003). Temporal components of the motor patterns expressed by the human spinal cord reflect foot kinematics. *Journal of Neurophysiology*. 90(5), 3555-3565

Ivanenko, Y.P., Poppele, R.E., and Lacquaniti, F. (2006). Spinal cord maps of spatiotemporal alpha-motoneuron activation in humans walking at different speeds. *Journal of Neurophysiology*. 95(2), 602-618.

Katsuyama, N., Usui, N., and Taira, M. (2016). Activation of the Human MT Complex by Motion in Depth Induced by a Moving Cast Shadow. *PloS one* 11(9)**,** e0162555.

Lacquaniti, F., Bosco, G., Gravano, S., Indovina, I., La Scaleia, B., Maffei, V., et al. (2014). Multisensory integration and internal models for sensing gravity effects in primates. *BioMed Research International* 2014:615854

Lacquaniti, F., Bosco, G., Indovina, I., La Scaleia, B., Maffei, V., Moscatelli, A., et al. (2013). Visual gravitational motion and the vestibular system in humans. *Frontiers in Integrative Neuroscience* 7**,** 101.

Lopez, C., and Blanke, O. (2011). The thalamocortical vestibular system in animals and humans. *Brain Research Reviews* 67(1-2)**,** 119-146.

Lopez, C., Blanke, O., and Mast, F. (2012). The human vestibular cortex revealed by coordinate-based activation likelihood estimation meta-analysis. *Neuroscience* 212**,** 159-179.

Maffei, V., Indovina, I., Macaluso, E., Ivanenko, Y.P., Orban, G.A., and Lacquaniti, F. (2015). Visual gravity cues in the interpretation of biological movements: neural correlates in humans. *Neuroimage* 104**,** 221-230.

Maffei, V., Mazzarella, E., Piras, F., Spalletta, G., Caltagirone, C., Lacquaniti, F., et al. (2016). Processing of visual gravitational motion in the peri-sylvian cortex: evidence from brain-damaged patients. *Cortex* 78**,** 55-69.

Nielsen, J.B. (2003). How we walk: central control of muscle activity during human walking. *The Neuroscientist* 9(3)**,** 195-204.

O'Connor, S.M., and Donelan, J.M. (2012). Fast visual prediction and slow optimization of preferred walking speed. *Journal of neurophysiology* 107(9)**,** 2549-2559.

Orlovsky, G. (1972a). Activity of vestibulospinal neurons during locomotion. *Brain Research* 46**,** 85-98.

Orlovsky, G. (1972b). The effect of different descending systems on flexor and extensor activity during locomotion. *Brain Research* 40(2)**,** 359-372.

Park, J.L., Dudchenko, P.A., and Donaldson, D.I. (2018). Navigation in real-world environments: new opportunities afforded by advances in mobile brain imaging. *Frontiers in Human Neuroscience* 12.

Pearson, K.G. (2004). "Generating the walking gait: role of sensory feedback," in *Progress in Brain Research*. Elsevier), 123-129.
Pickle, N.T., Grabowski, A.M., Auyang, A.G., and Silverman, A.K. (2016). The functional roles of muscles during sloped walking. *Journal of Biomechanics* 49(14)**,** 3244-3251.
Prochazka, A., and Ellaway, P. (2012). Sensory systems in the control of movement. *Compr Physiol* 2(4)**,** 2615-2627.
Regenbogen, C., Seubert, J., Johansson, E., Finkelmeyer, A., Andersson, P., and Lundström, J.N. (2018). The intraparietal sulcus governs multisensory integration of audiovisual information based on task difficulty. *Human Brain Mapping* 39(3)**,** 1313-1326.
Ryczko, D., and Dubuc, R. (2017). Dopamine and the brainstem locomotor networks: from lamprey to human. *Frontiers in Neuroscience* 11**,** 295.
Sherman, D., Fuller, P.M., Marcus, J., Yu, J., Zhang, P., Chamberlin, N.L., et al. (2015). Anatomical location of the mesencephalic locomotor region and its possible role in locomotion, posture, cataplexy, and parkinsonism. *Frontiers in Neurology* 6**,** 140.
Snaterse, M., Ton, R., Kuo, A.D., and Donelan, J.M. (2011). Distinct fast and slow processes contribute to the selection of preferred step frequency during human walking. *Journal of Applied Physiology* 110(6)**,** 1682-1690.
Sun, H.-J., Campos, J.L., and Chan, G.S. (2004). Multisensory integration in the estimation of relative path length. *Experimental Brain Research* 154(2)**,** 246-254.
Zhao, H., and Warren, W.H. (2015). On-line and model-based approaches to the visual control of action. *Vision Research* 110**,** 190-202.
